# Disturbed Flow Enhances Inflammatory Signaling and Atherogenesis by Increasing Thioredoxin-1 Level in Endothelial Cell Nuclei

**DOI:** 10.1371/journal.pone.0108346

**Published:** 2014-09-29

**Authors:** Young-Mi Go, Dong Ju Son, Heonyong Park, Michael Orr, Li Hao, Wakako Takabe, Sandeep Kumar, Dong Won Kang, Chan Woo Kim, Hanjoong Jo, Dean P. Jones

**Affiliations:** 1 Division of Pulmonary, Allergy and Critical Care Medicine, Department of Medicine, Emory University, Atlanta, Georgia, United States of America; 2 Division of Cardiology, Department of Medicine, Emory University, Atlanta, Georgia, United States of America; 3 Department of Molecular Biology, Dankook University, Yongin-si, South Korea; 4 Wallace H. Coulter Department of Biomedical Engineering, Georgia Institute of Technology and Emory University, Atlanta, Georgia, United States of America; Goethe Universität Frankfurt, Germany

## Abstract

**Background:**

Oxidative stress occurs with disturbed blood flow, inflammation and cardiovascular disease (CVD), yet free-radical scavenging antioxidants have shown limited benefit in human CVD. Thioredoxin-1 (Trx1) is a thiol antioxidant protecting against non-radical oxidants by controlling protein thiol/disulfide status; Trx1 translocates from cytoplasm to cell nuclei due to stress signaling, facilitates DNA binding of transcription factors, e.g., NF-κB, and potentiates inflammatory signaling. Whether increased nuclear Trx1 contributes to proatherogenic signaling is unknown.

**Methodology/Principal Findings:**

*In vitro* and *in vivo* atherogenic models were used to test for nuclear translocation of Trx1 and associated proinflammatory signaling. Disturbed flow by oscillatory shear stress stimulated Trx1 nuclear translocation in endothelial cells. Elevation of nuclear Trx1 in endothelial cells and transgenic (Tg) mice potentiated disturbed flow-stimulated proinflammatory signaling including NF-κB activation and increased expression of cell adhesion molecules and cytokines. Tg mice with increased nuclear Trx1 had increased carotid wall thickening due to disturbed flow but no significant differences in serum lipids or weight gain compared to wild type mice. Redox proteomics data of carotid arteries showed that disturbed flow stimulated protein thiol oxidation, and oxidation was higher in Tg mice than wild type mice.

**Conclusions/Significance:**

Translocation of Trx1 from cytoplasm to cell nuclei plays an important role in disturbed flow-stimulated proatherogenesis with greater cytoplasmic protein oxidation and an enhanced nuclear transcription factor activity. The results suggest that pharmacologic interventions to inhibit nuclear translocation of Trx1 may provide a new approach to prevent inflammatory diseases or progression.

## Introduction

Blood flow generates shear stress on vascular endothelial cells (EC) and regulates endothelial biology and cardiovascular disease (CVD), including atherosclerosis. Atherosclerotic lesions develop at arterial bifurcations and branch points that are exposed to patterns of disturbed or oscillatory blood flow, whereas straight arterial segments exposed to uni-directional, laminar flow are atherosclerosis-resistant [1], [2], [3]. Disturbed flow potently results in abnormal endothelial morphology and function including elevation of cell death, inflammation and thrombotic responses [4], [5]. Flow-sensitive patterns of gene expression have been identified including the upregulation of inflammatory genes [5], [6], [7] under conditions of disturbed flow, while laminar shear stress induces the expression of antiatherogenic, antiinflammatory and antioxidant genes [8], [9]. However, the molecular mechanisms by which EC detect local flow conditions and convert these into different patterns of signaling responses are not fully identified.

Thioredoxin (Trx) containing a pair of redox-active cysteine (Cys) in its catalytic site is the major cellular oxidoreductase enzyme regulating cellular redox homeostasis [10], [11]. Trx redox system including Trx, Trx reductase, peroxiredoxin, and NADPH, is critically involved in defense against oxidative stress, which has been implicated in the progression of most cardiovascular disease. Trx-1 is mostly localized in the cytoplasm, but its presence in cell nuclei is well known and differential functions in these compartments have been described. For instance, during cell stress induced by nutrient deprivation, proinflammatory signals, oxidants or reactive electrophiles, the nuclear Trx1 is more resistant to oxidation or depletion [12], [13], [14]. Trx1 translocates into nuclei from cytoplasm upon stress signals, e.g. H_2_O_2_, NO, UV, viral infection, and cadmium [15], [16], [17]. This translocation is critical for activation of transcription factors including NF-κB [18], [19], AP-1 [20], [21], [22], HIF-1α [23], Nrf-2 [24], [25], and p53 [26] that contain a regulatory Cys in the DNA binding region.

Compartmental regulation of NF-κB, AP-1 and Nrf-2 involves opposing redox-sensitive steps in cytoplasm and nuclei, i.e., upstream cytoplasmic oxidative activation involves kinase signaling, and downstream stimulation of DNA binding activity occurs through Trx1-dependent reduction of Cys in the DNA-binding domain [20], [27], [28]. For example, oxidative signaling in the cytoplasm initiates NF-κB activation via I-κB kinase, which phosphorylates and leads to degradation of I-κB causing dissociation and release of NF-κB for translocation into the nucleus [29]. In the nuclei, excessive oxidant production oxidizes a critical Cys residue (Cys^62^) in the DNA binding region of p50 NF-κB and inhibits DNA binding [19], [27]. In addition, Cys^38^ of p65 NF-κB plays a crucial role in DNA binding [30]. Increased nuclear Trx1 by transient transfection enhances DNA binding and increases NF-κB reporter activity [17], [18], [31] suggesting that the nuclear activation by Trx1 counters an endogenous H_2_O_2_-dependent transcriptional termination mechanism.

Modulation of NF-κB signaling by nuclear Trx1 raises the possibility that excessive nuclear Trx1 could cause a proinflammatory response accompanying hyper-responsive immune signaling. Indeed, our previous study using a transgenic mouse model in which nuclear Trx1 was increased due to expression of a fusion protein containing human Trx1 with a nuclear localization signal (NLS) showed that NF-κB activity is enhanced by nuclear Trx1 [17], [31]. NLS-Trx1 Tg mice with H1N1 influenza infection had greater inflammatory response, including elevated NF-κB activity and IL-6 and TNF-α induction [31]. In a recent study, we also found that environmental low level Cd exposure stimulated Trx1 nuclear translocation and NF-κB activation in lung fibroblasts [17]. Thus, potentiation of atherogenic signaling by nuclear Trx1 could represent a general mechanism for multiple stressors contributing to CVD.

In the present study, we examined the role of nuclear Trx1 in atherogenic signaling using mouse and cultured EC models. The results from an atherogenic mouse model show that increased Trx1 in cell nuclei stimulated disturbed flow-induced carotid wall thickening, activated NF-κB and elevated VCAM level with no significant increase in mouse weight gain or blood lipid levels. Proteins in the carotid artery were substantially oxidized by disturbed flow and this oxidation was enhanced by nuclear overexpression of Trx1. Transient overexpression of Trx1 in cell nuclei of EC potentiated OS-dependent NF-κB activation and elevated proinflammatory cytokine and cell adhesion molecule genes. Together, the data show that increased nuclear Trx1 causes disruption of nuclear function that potentiates atherogenic signaling and represents a potential target for therapeutic intervention.

## Materials and Methods

### Ethics Statement

This study was carried out in strict accordance with the recommendations in the Guide for the Care and Use of Laboratory Animals of the Emory University. All protocols involving mice in this study were reviewed and approved by the Emory University Institutional Animal Care and Use Committee (IACUC).

### Cell culture, transfection, and shear stress

Human umbilical vein endothelial cells (HUVECs) were purchased from Lonza (Basel, Switzerland), cultured in M199 media (Cellgro, Manassas, VA) with 20% fetal bovine serum (FBS, Atlanta Biologicals) and used between passages 5 and 6 on 0.1% gelatin-coated 100-mm dishes at 37°C and 5% CO_2_ as described previously [32], [33]. To investigate effects of nuclear Trx1 *in vitro*, cells were transiently transfected with NLS-Trx1 in nuclei [31] or vector control using HUVEC Nucleofector Kit (Lonza) as per manufacture's recommendations and allowed to recover for 48 h. HUVEC one-day post-confluence were exposed to unidirectional laminar shear (LS, 15 dyn/cm2) or oscillatory shear (OS, ±5 dyn/cm2 at 1 Hz frequency) for 24 hours using a cone-and-plate device [32], [33].

### Subcellular fractionation and western blotting

To examine Trx1 nuclear translocation by shear stress, subcellular fractionation of endothelial cells exposed to shear stress was performed using Qproteome kit (Qiagen, Valencia, CA) following the procedures provided by the manufacturer. Isolated fractions were then confirmed by Western blotting probed with β-actin and lamin for cytoplasm and nuclei antibodies, respectively. Trx1 levels in these fractions were determined by Western blotting probed with Trx1 antibody (AbFrontier, Seoul, Korea). Alexa-Fluor-680-conjugated anti-rabbit or anti-mouse secondary antibody (Invitrogen) was used and a band corresponding to each protein was visualized using an Odyssey scanner and Odyssey 2.1 software (Li-Cor, Lincoln, NE).

### Quantitative real-time PCR (qPCR)

Total RNA was polyadenylated and reverse transcribed for use in a two-step qRT-PCR using the High-capacity cDNA Synthesis kit (ABI) and qRT-PCR kits (Stratagene) as described [34]. The resulting cDNA was subjected to qPCR using forward and reverse primers for the selected genes. A master mix was prepared for each PCR, which included SYBR Green qRT-PCR SuperMix, forward and reverse primer, ROX reference dye and template cDNA. The reactions were monitored using a preheated real-time instrument (ABI StepOne Plus). The PCR conditions were 10 min at 95°C, followed by 40 cycles of 95°C for 15 s and 60°C for 30 s. All qPCR results were normalized based on 18S RNA expression in each sample. Fold changes between different treatments were determined for all targets using the ΔCt method [35]. Sequences for primers used have been listed in Table 1. Primers were procured from Integrated DNA Technologies (Coralville, IA).

**Table 1:** Comparison of serum lipid levels and body weight between WT and NLS-Trx1 Tg mice after challenging with disturbed flow and high fat diet for 8 weeks.

	WT	NLS-Trx1 Tg
Body weight (g, 0 week)	18.8±0.8	19.4±0.7
Body weight (g, 8 week)	25.3±0.9	24.2±1.2
Cholesterol (mg/dL, 0 week)	77.3±7.7	69.7±7.4
Cholesterol (mg/dL, 8 week)	201.0±11.8	214.7±22.8
Triglyceride (mg/dL, 0 week)	54.3±5.4	56.3±3.2
Triglyceride (mg/dL, 8 week)	95.0±8.7	97.7±10.4
HDL (mg/dL, 0 week)	44.0±5.9	37.9±5.3
HDL (mg/dL, 8 week)	44.9±3.9	56.9±6.6
LDL (mg/dL, 0 week)	4.9±2.1	2.7±2.5
LDL (mg/dL, 8 week)	83.9±6.9	81.9±9.9

### Animal studies with partial carotid ligation

Transgenic male mice (8 weeks of age) expressing human Trx1 in cell nuclei [NLS-Trx1 Tg [31]] and littermate wild type male (WT) mice were anesthetized with 3.5% isoflurane initially and then 1.5 to 2% during the entire procedure. Mice underwent partial ligation of the left carotid artery (LCA) as previously described [36], [37], [38]. In short, the surgical site was epilated, disinfected with betadine, and a ventral mid-line incision (4 to 5 mm in length) was made in the neck using micro-scissors. The LCA bifurcation was exposed by blunt dissection and three of four caudal LCA branches (left external carotid, internal carotid, and occipital arteries) were carefully dissected free of surrounding connective tissue and ligated with 6–0 silk sutures, leaving the superior thyroid artery intact. The contralateral right carotid artery (RCA) was left intact as an internal control. The surgical incision was then closed with 6–0 monofilament sutures and Tissue-Mend (Veterinary Product Laboratories), and analgesic buprenorphine (0.1 mg/kg) was administrated subcutaneously. The mice were monitored until recovery in a chamber on a heating pad. Following carotid ligation, disturbed blood flow (*d-flow*) in LCA and stable flow (*s-flow*) in RCA was confirmed at one day post-ligation by Doppler ultrasonography using the Vevo770 system (Visualsonics, Toronto, Canada) with a 30-MHz probe (RMV707B) [36], [37]. For redox proteomics and en face immunostaining studies, mice were maintained on standard chow diet following ligation. For functional tests of arterial wall thickening, mice were maintained for 4 weeks on the Paigen's high-fat diet (HFD; Science Diets, Topeka, KS) containing 1.25% cholesterol, 15% fat, and 0.5% cholic acid [39] following ligation. All animal studies were carried out by procedures approved by the Emory University Institutional Animal Care and Use Committee (IACUC).

### Arterial wall thickening assessment

For studies of carotid arterial wall thickening, ligated mice fed a HDF for 4 weeks were euthanized by CO_2_ inhalation and perfused with saline containing heparin. Frozen section tissue sample preparation and Oil red O (O-R-O) staining were performed as described previously [36]. Briefly, LCA and RCA were collected en block with the heart, aortic arch, trachea, esophagus, and tissue. Frozen sections were made starting from the level of the right subclavian artery bifurcation, 300 µm was trimmed away and three sets of ten consecutive 7 µm thick sections were taken at 300 µm intervals constituting the ‘proximal’ and ‘middle’ portions of the artery. Samples were imaged using an inverted microscope (IX71, Olympus, Japan) at 10x magnification. Images were analyzed with NIH Image J software to quantify wall thickness in each animal as described above.

### En face immunofluorescent staining

Mice were euthanized by CO_2_ inhalation and perfused with saline containing heparin, followed by a second perfusion with 10% formalin. Arteries were carefully cleaned in situ, dissected free of surrounding fat tissue, fixed in 4% paraformaldehyde, and tissue samples from LCA and RCA. Samples were en face stained with NF-κB p65 antibody (Cell Signaling Technology, Boston, MA) and VCAM-1 antibody (BD Pharmingen, San Jose, CA) as described previously [7]. En face images were collected with a Zeiss LSM 510 META confocal microscope (Carl Zeiss, Germany).

### Mass spectrometry based redox proteomics

Redox ICAT was performed using Isotope Coded Affinity Tag (ICAT)-based mass spectrometry [40], [41], [42], [43], [44], [45]. Twelve mice for each WT and NLS-Tg (6–8 weeks) were ligated as described above. The carotid arteries were collected at 48 h post ligation, and then total arterial protein samples were extracted from RCA and LCA (12 carotids pooled) of each WT and NLS-Tg, respectively. Briefly, RCA and LCA were pulverized in the liquid nitrogen and then proteins were immediately precipitated with 10% TCA. Protein precipitate was washed with ice-cold acetone, resuspended in denaturing buffer provided by the manufacturer and treated with the biotin-labeled thiol reagent [Heavy isotopic (H-ICAT)] for 1 h at 37°C. Unlabeled oxidized forms (e.g., disulfides) in the proteins were then reduced by TCEP [tris-(2-carboxyethyl phosphine)] and finally labeled with the second biotin-labeled thiol reagent [Light isotopic (L-ICAT)] for 1 h. Samples were digested with trypsin for 18 h, fractionated by cationic exchange followed by avidin purification, and analyzed by mass spectrometry as described previously [40]. Peptides were identified with an H to L ratio as a measure of the reduced/oxidized state of protein, expressed as percentage values, and labeled as “% oxidized state”. Identified peptides were individually processed to eliminate redundancies, and matched to proteins based upon amino acid sequences [45].

### Statistics

Statistical comparisons of data were carried out using the t-test of the OriginLab (Data Analysis and Graphing software, OriginLab Co.). P<0.05 was considered to be significant.

## Results

### Increased Trx1 expression in cell nuclei potentiates carotid wall thickening by partial ligation-induced disturbed flow

**Figure 1:**
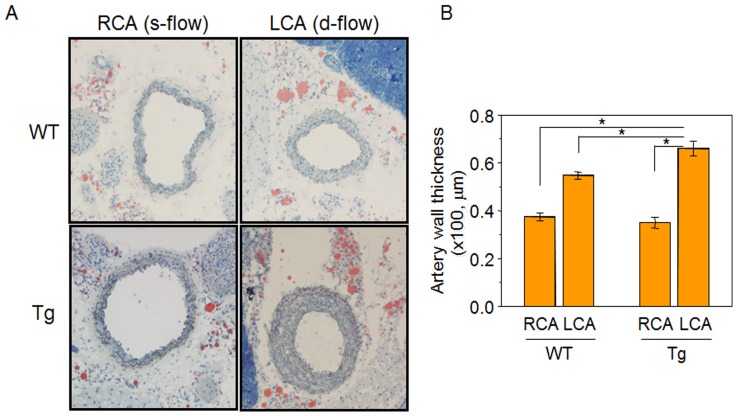
Enhanced carotid wall thickening by increased nuclear Trx1 in NLS-Trx1 Tg mice. The 11 mice for each WT and NLS-Trx1 Tg were partially ligated in left carotid artery [LCA, disturbed (d)-flow] while RCA [steady (s)-flow] was left intact and examined for carotid wall thickening by histology using Oil Red O staining on frozen sections (A). A bar graph (B) shows wall thickness measured using NIH image J software. Data are means ± SE (* p<0.05).

To determine the effect of nuclear Trx1 on disturbed flow induced atherosclerosis development, changes in carotid wall thickening were examined in NLS-Trx1 Tg and WT littermate exposed to either disturbed-flow (d-flow, LCA region) or steady laminar flow (s-flow, RCA region). Consistent with the previous studies [37], [38], increased carotid wall thickening was observed in both WT and NLS-Trx1 Tg mice due to ligation-induced disturbed flow. However, this increment was substantially higher in LCA of NLS-Trx1 Tg than WT [WT-RCA (37.4±1.6 µm), WT-LCA (54.8±1.4 µm), Tg-RCA (35.0±2.3 µm), Tg-LCA (66.0±3.0 µm)] (Fig. 1) and also Oil red O staining showed evidence of increased fat accumulation contributing to atherosclerosis development in Tg compared to WT mice.

### Potentiation of atherogenesis in NLS-Trx1 Tg mouse challenged with disturbed flow is not associated with increases in body weight or total lipid levels

To determine whether potentiation of atherogenic events, increased wall thickening observed in NLS-Trx1 Tg by disturbed flow, was associated with other risk factors for atherosclerosis such as high cholesterol level and obesity, WT and Tg mice that underwent LCA ligation and fed high fat diet for 8 weeks were examined for lipid levels and body weight. Results showed that there was no significant difference between WT and Tg in body weight or total serum lipid levels at baseline (0 week) or following 8 weeks (Table 1).

### Nuclear Trx1 stimulates p65 NF-κB expression and activation, and VCAM1 expression by disturbed flow

**Figure 2:**
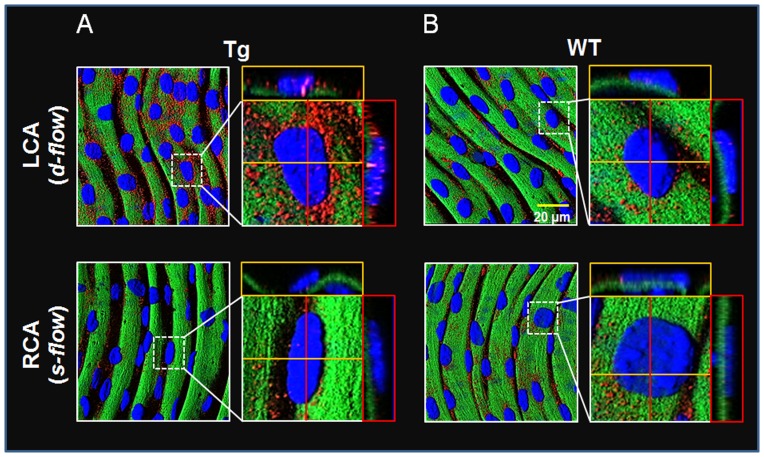
Potentiation of disturbed flow-stimulated NF-κB activation by nuclear Trx1. WT and NLS-Trx1 Tg mice ligated in LCA were examined for p65 NF-κB expression and nuclear localization using *en face* fluorescence confocal examination (red, p65 NF-κB; green, elastic layer; blue, DAPI; magenta, overlapping DAPI and p65).

**Figure 3:**
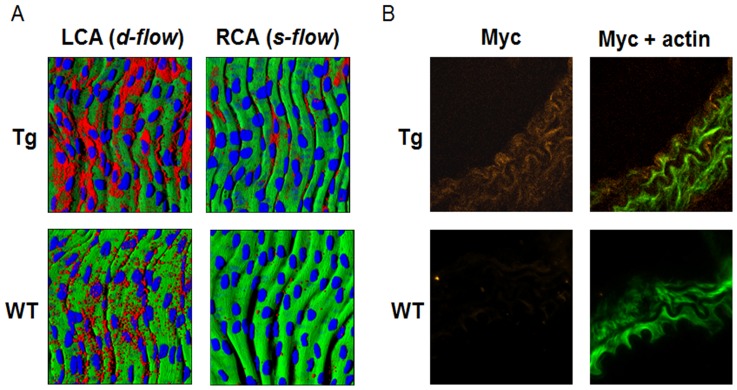
Potentiation of disturbed flow-stimulated VCAM1 by nuclear Trx1. A) Frozen sections of LCA and RCA from NLS-Trx1 Tg and WT littermate mice were examined for VCAM1 expression by *en face* fluorescence confocal examination (red, VCAM1; blue, nucleus; green, elastic layer). B) LCA of WT and Tg were examined for NLS-Trx1 (Myc epitope tag) expression using immunofluorescence staining on frozen section by probing with an antibody against Myc (orange, Myc; green, actin phalloidin).

Activation of NF-κB plays a key role in regulating proinflammatory signaling in association with elevated expression of cytokine and cell adhesion molecules. To determine whether stimulation of disturbed flow-induced atherogenic signaling is associated with NF-κB activation, p65 NF-κB and VCAM1 were examined in WT and Tg mice using *en face* staining of carotid arteries. The results showed that p65 expression was substantially increased in LCA of Tg (Fig. 2A top) by disturbed flow compared to that in LCA of WT (Fig. 2B top, red; p65, blue; dapi). Moreover, elevated p65 nuclear translocation was observed in LCA of Tg suggesting that disturbed flow-induced NF-κB activation was potentiated by Trx1 in nuclei (Fig. 2A top, magenta; p65 in nuclei). Consistently, Trx1 in nuclei potentiated VCAM1 expression in LCA exposed to disturbed flow (Fig. 3A). To verify potentiation of proinflammatory signaling is associated with NLS-Trx1, LCA of Tg and WT was examined by immunofluorescence probed with anti-Myc antibody to detect the Myc epitope in the Myc-tagged NLS-Trx1. As expected, Myc fluorescence was only observed in Tg but not in WT, confirming NLS-Trx1 expression in Tg (Fig. 3B).

**Figure 4:**
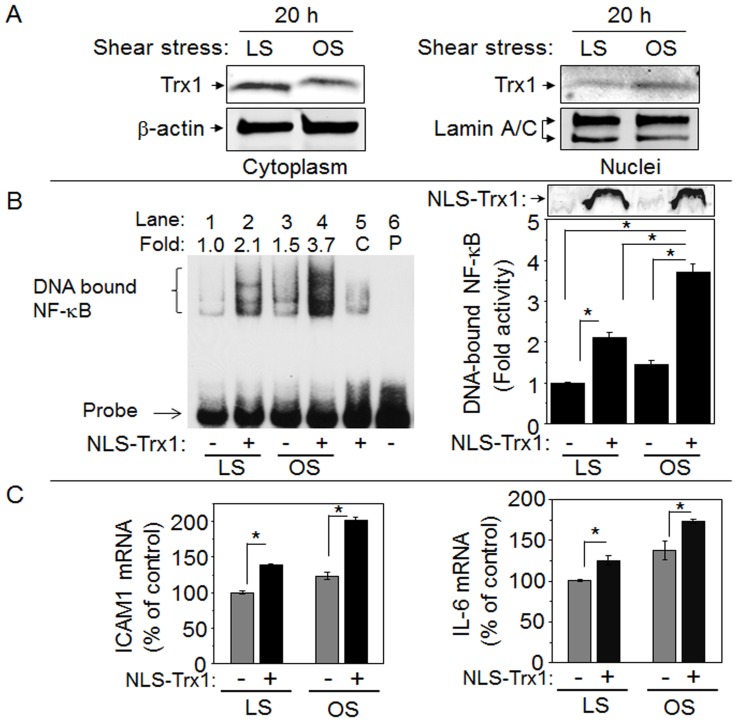
Nuclear translocation of Trx1 in response to oscillatory shear stress and stimulated NF-κB activation and proinflammatory gene expression by increased nuclear Trx1. EC exposed either LS or OS for 24 h were fractionated to obtain cytosolic and nuclear fractions. A. Cytosolic (left) and nuclear (right) fractions were examined for Trx1 expression level by Western blotting probed with an antibody specific to Trx1. To verify each compartment, cytosolic and nuclear fractions were probed with β-actin and lamin antibodies, respectively. B. EC transfected with NLS-Trx1 or VC were exposed to LS or OS for 24 h and nuclear fractions were examined for NF-κB activity by EMSA. NLS-Trx1 expression measured by Western blotting with antibody specific to Myc epitope is shown on right, top. C. Total RNA was isolated from EC exposed to LS or OS after NLS-Trx1 transfection. cDNA obtained from reverse transcription of total RNA were quantitated for ICAM1 and IL-6 genes by qRT- PCR. Data are mean ± SE (* *p*<0.05).

To support *in vivo* results that nuclear Trx1 plays a pivotal role in potentiating disturbed flow-induced proinflammatory signaling, we performed *in vitro* shear experiments using a cone and plate device to generate disturbed oscillatory (OS) shear stress with laminar shear (LS) as a control. Endothelial responses are different to OS and LS, with OS inducing proinflammatory and proatherogenic signaling and LS inducing antiatherogenic signaling. Such responses of EC to LS and OS were confirmed by examining EC morphology; EC alignment was observed from LS exposure for 20 h but not from OS (data not shown). In addition, OS stimulated decrease in Iκ-Bα compared to LS, consistent with OS-stimulated NF-κB activation (data not shown). After confirming these well-recognized OS and LS effects, we examined Trx1 nuclear translocation. Cytoplasm and nuclei compartments were examined for Trx1 together with β-actin and lamin, respectively, to confirm each fraction (Fig. 4A). The result showed that Trx1 was higher in nuclei of EC exposed to OS than LS for 20 h. Trx1 in cytoplasm was lower by OS than LS, suggesting that OS, like other types of stressors, stimulates nuclear translocation of Trx1.

To examine consequences of increased nuclear Trx1, Trx1 was transiently overexpressed in nuclei by transfecting HUVEC with a plasmid expressing NLS-Trx1 (same plasmid used to create NLS-Trx1 Tg mice) [17], [31]. 2 d after transfection, EC were exposed to either LS or OS, and nuclear fractions were isolated and examined by EMSA for NF-κB activation (Fig. 4B left and right). A bar graph (right) shows the NF-κB activity quantified from intensity of DNA bound NF-κB (left). The results showed that OS stimulated NF-κB activation 1.5-fold compared with LS (Fig. 4B, lane 1 and 3, without NLS-Trx1) consistent with decreased Iκ-B resulting from OS. OS-stimulated NF-κB activity was elevated 3.7 fold with expression of NLS-Trx1 (Fig. 4B, lane 4, with NLS-Trx1) supporting the role of nuclear Trx1 in potentiation of OS-induced NF-κB activation. Elevation of Trx1 expression in EC nuclei was confirmed by anti-Myc (Fig. 4B right, top panel, with NLS-Trx1). To test for effects of nuclear Trx1 on proinflammatory transcripts under transcriptional control of NF-κB, ICAM1 and IL-6 expression were examined by real time PCR. Consistent with substantial elevation of VCAM1 expression in Tg mouse with disturbed flow, OS-induced ICAM1 and IL-6 expression were significantly elevated by increased nuclear Trx1 (Fig. 4C). Similarly, this potentiation effect of nuclear Trx1 on NF-κB controlled-proinflammatory cytokines including IL-6 was also shown in our previous study of H1N1 influenza virus infection [31]. Increased mRNA levels of ICAM1 and IL-6 together with increased levels of VCAM1 and p65 NF-κB, and increased activity of NF-κB support the key finding that nuclear Trx1 potentiated disturbed flow-induced inflammatory signaling.

### Disturbed flow stimulates protein oxidation

**Figure 5:**
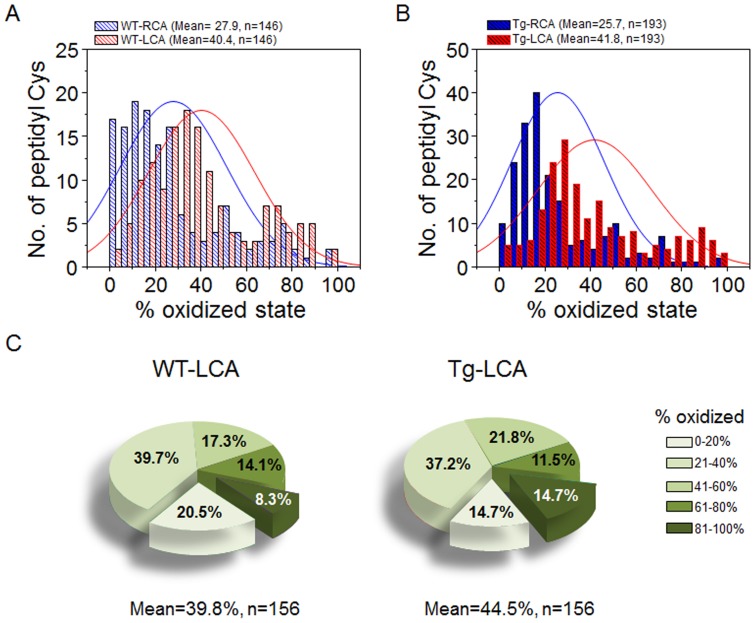
Distribution of oxidized state of peptidyl Cys in RCA and LCA of WT and NLS-Trx1 Tg mice. Histograms (A, B) show numbers of peptidyl Cys identified by redox ICAT/MS according to % oxidation measured in RCA and LCA of each WT (A) and NLS-Trx1 Tg (B) mice. C, Pie charts show the distribution of oxidized peptidyl Cys of WT-LCA and Tg-LCA. *P*-values analyzed by Chi-square test comparing RCA and LCA from WT (A), RCA and LCA from Tg (B), and subset of peptides (C) from LCA of WT and Tg are as follows: RCA (WT) vs. LCA (WT), *p* = 9.53×10^−31^; RCA (Tg) vs. LCA (Tg), *p* = 1.37×10^−55^, LCA (WT) vs. LCA (Tg), *p* = 0.0225.

Previous research shows that disturbed flow causes oxidative stress, which we confirmed with measures of the GSH system. Under the conditions studied, mixed disulfide of GSH and Cys forms as the primary product of GSH oxidation [46] and was higher in artery (LCA) exposed to disturbed flow than RCA (LCA:RCA, 2.1∶1, n = 3). Moreover, fold increase of the mixed disulfide in LCA compared to RCA was even higher (LCA:RCA, 4.6∶1, n = 3) in NLS-Trx1 Tg. We therefore used these conditions to study the effect of disturbed flow on protein oxidation. Mass spectrometry-based redox ICAT technique was used to investigate the effect of disturbed flow on carotid artery protein redox state in 12 mice each for WT and Tg following ligation surgery in LCA. Carotid aortic tissues were collected 48 h after the ligation procedure. Results for WT mice showed that average oxidation of cysteine (Cys) residues of proteins was greater in LCA, 40.4% compared to RCA, 27.9% (Fig. 5A), showing that disturbed flow induces protein oxidation consistent with regional oxidative stress. Average % oxidation data for peptidyl Cys and respective proteins identified from WT mice are included in Supporting Information (Table S1). Table S1 shows that the distinct consequence of increased oxidation of proteins and peptidyl Cys by disturbed blood flow due to partial carotid ligation appear to affect cytoskeleton remodeling and cell proliferation signaling mechanisms. Proteins and peptidyl Cys associated with these mechanisms include actin, actin-associated molecules such as destrin (actin depolymerizing factor), ras, smoothelin, latent transforming growth factor binding protein, and eukaryotic translation elongation factors (Table S1).

A similar pattern of oxidation was observed for NLS-Trx1 Tg mice [LCA, 41.8%; RCA, 25.7% (Fig. 5B)], with evidence supporting potentiation of ligation-induced protein oxidation by increased Trx1 in nuclei. Supporting Information (Table S2) includes average % oxidation data of peptidyl Cys and respective proteins identified from Tg mice. For instance, redox values of LCA between WT and Tg, 156 peptidyl Cys identified in both WT- LCA and Tg- LCA were analyzed according to the measured percent oxidation (Fig. 5C). The data showed that proteins of Tg- LCA (44.5%) were more oxidized than WT LCA (39.8%), consistent with more extensive proatherogenic response. Results showed that 20.5% of peptides were <20% oxidized in WT, while 14.7% of peptides were <20% oxidized in Tg, and 8.3% of peptides were >81% oxidized in WT, while 14.7% of peptides were >81% oxidized in Tg. Average % oxidation data for all peptidyl Cys and respective proteins for this analysis are shown in Supporting Information (Table S3). The results showed that average oxidation of proteins in Tg- LCA was greater than WT- LCA, supporting the proinflammatory role of NLS-Trx1 shown above (Fig. 1–4).

## Discussion

The present study shows that disturbed oscillatory shear stress induces translocation of Trx1 into vascular endothelial cell nuclei and that increased nuclear Trx1 potentiates proinflammatory signaling by NF-κB and atherogenesis in a mouse model of CVD. Earlier studies show that Trx redox state is controlled differently in subcellular compartments. The mitochondria are the most reduced compartment and mitochondrial Trx2 is highly sensitive to oxidation [47], [48]. In contrast, nuclei are also maintained as relatively reduced redox state, but nuclear Trx1 is resistant to oxidation [14], [48], [49]. While diverse studies have indicated important roles for Trx in modulating vascular injury, including oxidation of mitochondrial Trx2, no previous studies have determined the role of nuclear Trx in atherosclerosis development.

The present studies show that redox regulation of NF-κB activation due to increased nuclear Trx1 has an important role in potentiating vascular pathophysiology in the mouse partial carotid ligation model of atherogenesis. The results with the NLS-Trx1 Tg mouse showed that NF-κB activity was increased, transcripts for cell adhesion molecules were increased, and significant carotid artery wall thickening and increased Oil-Red-O staining were evident at the region exposed to disturbed blood flow. In these studies with increased nuclear Trx1, no detectable changes in serum lipid levels or weight gain was observed, indicating that the proatherogenic effects observed in NLS-Trx1 Tg mouse were not due to indirect effects on lipid metabolism. In comparative studies (not shown), we also examined effects of overexpression of Trx1 in cytoplasm using a transgenic mouse line expressing Trx1 fusion protein containing a nuclear export signal and Trx2 in mitochondria using a transgenic mouse line expressing human Trx2 and observed no stimulation of inflammation or proatherogenic signaling. Thus, the *in vivo* studies with this well-established partial carotid ligation model for CVD clearly show an important contribution of increased nuclear Trx1 to the known pathophysiologic pathway.

**Figure 6:**
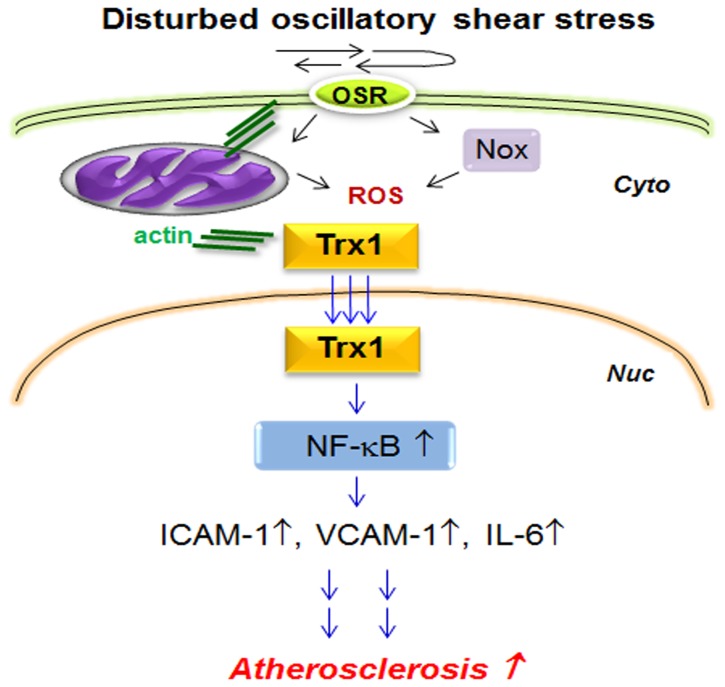
Proposed scheme for nuclear Trx1 in atherosclerosis. Disturbed oscillatory shear stress stimulates ROS production by Nox and mitochondria, resulting in altered protein redox state and change in actin cytoskeleton structure. Changes in actin structure result in translocation of Trx1 into nuclei. Increased nuclear Trx1 potentiates proinflammatory signaling by activating redox sensitive transcription factor NF-κB. Increased activity of NF-κB results in increased abundance of cell adhesion molecules and inflammatory cytokines contributing to atherogenesis.

The *in vivo* results were confirmed *in vitro*; overexpression of Trx1 in HUVEC nuclei showed potentiation of NF-κB activation and elevation of cell adhesion molecule and cytokine (ICAM1, IL-6) by OS. In addition to NF-κB regulation, elevated Trx1 in nuclei resulted in increased mRNA expression of Bax and procaspase-3 but decreased Bcl-2 (data not shown). These genes are closely associated with activation of cell death and regulated by HIF1α and AP-1 transcription factors [50] that are redox sensitively regulated by Trx1. This result further supports potentiation of OS-induced proinflammatory and cell death signaling by nuclear Trx1 via activating redox-sensitive transcription factors. The results from the current study enable an outline of the signaling scheme for a pathologic role of nuclear Trx1 in proinflammatory/proatherogenic events (Fig. 6).

Shear stress is mechanotransduced into a biochemical signal that results in changes in vascular behavior. Maintenance of steady laminar blood flow is crucial for normal healthy vascular function while disturbed oscillatory flow near arterial bifurcations and curvatures is associated with atherosclerosis. In addition, vascular endothelial cells respond differently to different shear flow at the molecular and cellular levels, and such responses are associated with opposite consequences either preventing or promoting atherosclerosis. In the present study, *in vitro* data also show differential responses of EC to LS and OS. However, it is still largely unknown how OS and LS induce signaling differently ultimately resulting in opposite cellular responses, atheroprotection and atheropromotion. Therefore, further studies with systematic approaches are required, for instance, identification of the source for oxidant generation (mitochondria, NADPH oxidases) because redox signaling is involved in both LS and OS responses. This study will be needed to provide information for Trx1 nuclear translocation mechanism since previous studies [16], [17], [31] and the current finding suggest that oxidative stress controls Trx1 nuclear translocation even though the nuclear import mechanism occurs through targeting lysine residues [16].

Shear stress regulates endothelial cell alignment and structure by controlling actin cytoskeletal reorganization [51] and this remodeling was regulated by redox-dependent manner [52]. Our previous studies also show that actin polymerization is controlled in a redox-sensitive manner [42], [43]. The study by Zschauer showed that H_2_O_2_-stimulated endothelial cell death was prevented by direct interaction of Trx1 with actin [53]. This event appeared to be associated with changes in actin cytoskeleton redox states. Oxidation and chemical modifications of Cys residues are closely associated with pathologic mechanisms [54]. A small number of studies provide evidence that modification of specific Cys can be mechanistically important. For instance, microinjection of EC with modified Trx1 at Cys^73^ residue by acrolein, resulted in stimulation of monocyte adhesion, an early step in atherogenesis [55]. To obtain a more global understanding of physiologic and pathophysiologic protein oxidation, we developed redox proteomic method to measure the fractional oxidation of specific Cys residues in proteins [40], [41], [42], [45]. Using this technique, we identified a subset of proteins in carotid arteries that are sensitive to oxidation and associated with atherogenic mechanism [see Supporting Information (Table S1–S3)]. Importantly, a number of actin cytoskeleton proteins were oxidized due to partial ligation-induced disturbed blood flow (Fig. 5A, Table S1), and increased Trx1 level in nuclei potentiated oxidation of these proteins (Fig. 5B, Table S2, S3), supporting actin cytoskeleton function associated with Trx1 redox regulation system [17]. In addition, our previous studies with mouse aortic endothelial cells, we found that increased extracellular cystine (CySS) oxidized form of Cys, which has been associated with adverse outcomes in patients with acute coronary syndrome and other vascular dysfunction [56], [57], [58], increased mitochondrial oxidant production and caused oxidation of multiple actin-associated proteins [43]. Although requiring further detailed investigation, the results suggest convergent mechanisms whereby mitochondrial or Nox-derived oxidation of actin-cytoskeletal proteins results in increased nuclear Trx1 content and potentiation of atherogenic events.

As shown in our previous study [31], increased nuclear level of Trx1 did not have an effect on other major cellular and extracellular redox systems GSH/GSSG and Cys/CySS redox states, respectively, and expression levels of antioxidant molecules including mitochondrial Trx2, peroxiredoxin (Prx)-1, Prx2 and Prx3 [31]. On the other hand, localized exposure to disturbed flow in carotid artery region resulted in oxidation of multiple proteins compared to the regions exposed to stable blood flow (LCA vs. RCA). This result is consistent with the previous study showing that GSH/GSSG redox state of endothelial cells exposed to oscillatory shear stress was substantially oxidized by decreasing cellular GSH level while laminar shear stress had an opposite effect on GSH level [59].

In summary, the current study shows that increased Trx1 in cell nuclei potentiates atherogenesis. The results show that oscillatory shear induced nuclear translocation of Trx1 and that increased Trx1 caused enhanced carotid wall thickening, lipid accumulation and proinflammatory signaling in a disturbed flow model of CVD. Redox proteomics showed that disturbed flow resulted in oxidation of actin cytoskeleton proteins in mouse carotid artery and this oxidation was elevated by increased nuclear Trx1. Taken together, the data show that nuclear translocation of Trx1 is a critical step of endothelial dysfunction induced by disturbed oscillatory flow. Because cytoplasmic oxidative stress and other stress signals stimulate nuclear translocation of Trx1 in different cell types, the results suggest that excessive nuclear Trx1 due to diverse stressors may be a general contributor to the prevalence of CVD. Consequently, the results suggest new therapeutic targets to modulate Trx1 nuclear translocation and compartmental redox systems to prevent or delay atherosclerosis.

## Supporting Information

Table S1 **Redox ICAT/MS-measured % oxidation of peptidyl Cys/protein of RCA and LCA in WT mice.** The data show 146 peptidyl Cys with % oxidation (%ox) value identified from RCA and LCA of WT mice.
(PDF)

Table S2 **Redox ICAT/MS-measured % oxidation of peptidyl Cys/proteins of RCA and LCA in NLS-Trx1 Tg mice.** The data show 193 peptidyl Cys with % oxidation (%ox) value identified from RCA and LCA of Tg mice.
(PDF)

Table S3 **Redox ICAT/MS-measured % oxidation of peptidyl Cys/proteins of LCA in WT and Tg mice.** The data show 156 peptidyl Cys with % oxidation (%ox) value identified from LCA of WT and Tg mice.
(PDF)
