# Supplementary material for: Disturbed Flow Enhances Inflammatory Signaling and Atherogenesis by Increasing Thioredoxin-1 Level in Endothelial Cell Nuclei
Source: PLoS One. 2014 Sep 29;9(9):e108346. doi: 10.1371/journal.pone.0108346 (PMC4180949; doi:10.1371/journal.pone.0108346)
Supplement: Table S1 — Redox ICAT/MS-measured % oxidation of peptidyl Cys/protein of RCA and LCA in WT mice. The data show 146 peptidyl Cys with % oxidation (%ox) value identified from RCA and LCA of WT mice. (PDF) [file pone.0108346.s001.pdf]

Table S1. Redox ICAT/MS-measured % oxidation of peptidyl Cys/protein of RCA and LCA in WT mice. The data show 146 peptidyl Cys with % oxidation (%ox) value identified from RCA and LCA of WT mice.

Peptide	Cys	Accession	Annotation	%ox RCA	%ox LCA
ACLISLGYDVENDR	C794	NP_068695.1	actinin alpha 4	21.9	36.6
ACPVGHHLHR	C864	NP_783572.2	latent transforming growth factor beta binding protein 4 isoform a	55.4	67.6
AGLSSGFVGCVR	C3248	XP_001477365.1	PREDICTED: similar to perlecan (heparan sulfate proteoglycan 2)	46.4	46.8
AHGQDLGTAGSCLR	C1493	NP_034061.2	collagen, type IV, alpha 1	61.8	75.1
AHNQDLGLAGSCLAR	C1532	NP_034062.2	procollagen, type IV, alpha 2	69.4	67.1
AHSVEECR	C888	XP_001477365.1	PREDICTED: similar to perlecan (heparan sulfate proteoglycan 2)	78.0	88.5
AHVAPCFDASK	C1157	NP_034357.2	filamin, alpha	3.3	29.2
ALANSLACQGK	C339	NP_031464.1	aldolase 1, A isoform	16.1	40.3
ALIQCAK	C950	NP_033528.3	vinculin	27.1	32.0
APSVANIGSHCDLSLK	C2152	NP_034357.2	filamin, alpha	18.5	26.7
ASPFIECHGR	C1615	NP_031760.2	procollagen, type IV, alpha 3	76.1	82.8
AVIFCLSADK	C39	NP_062745.1	destrin	32.4	58.5
CAELEEELK	C190	NP_077745.2	tropomyosin 1, alpha	0.6	7.6
CAPGVVGPTEADIDFDIIR	C810	NP_034357.2	filamin, alpha	10.3	40.4
CATSTPAFFAEK	C270	NP_034860.2	annexin A1	32.4	32.4
CAVVDVPFGGAK	C172	NP_032159.1	glutamate dehydrogenase 1	11.6	35.3
CDDLEALKK	C64	NP_034708.1	integrin beta 1 (fibronectin receptor beta)	65.3	82.5
CDKEVYFAER	C7	NP_031789.1	cysteine-rich protein 1 (intestinal)	30.7	42.4
CDRVDQLTAQLADLAAR	C545	NP_033528.3	vinculin	27.1	32.1
CDSGFALDSEER	C1057	NP_032019.2	fibrillin 1	36.4	68.1
CDVDIR	C286	NP_780706.1	actin, beta-like 2	24.2	40.5
CEKPFLGHR	C227	NP_659111.1	LIM and senescent cell antigen like domains 2	18.6	38.9
CGDSVYAAEK	C122	NP_031818.3	cysteine and glycine-rich protein 2	27.7	28.8
CGEVVQEHVIR	C248	NP_598515.3	filamin binding LIM protein 1	31.9	48.1
CGTGIVGVFVK	C261	NP_058557.2	carboxyl terminal LIM domain protein 1	35.9	29.5
CIESLIAVFQK	C8	NP_058020.1	S100 calcium binding protein A11 (calizzarin)	0.5	12.0
CIIPNHEKR	C678	NP_780469.1	myosin heavy chain 10, non-muscle	7.9	18.3
CILLSNLSNTSHAPK	C1464	NP_032014.3	fatty acid synthase	17.9	27.0
CLAFHDISPQAPTHFLVIPK	C38	NP_032274.1	histidine triad nucleotide binding protein 1	11.7	34.8
CLGIPTR	C290	NP_033399.1	transglutaminase 2, C polypeptide	12.6	9.1
CLHPLASETFVSK	C71	NP_034341.2	four and a half LIM domains 1 isoform 3	21.7	32.0

CLIEILASR	C111	NP_036052.2	annexin A10	1.1	7.9
CPETLFQPSFIGMESAGIHETTYNSIM*K	C259	NP_033738.1	actin, alpha, cardiac	2.9	38.9
CQLEINFNTLQTK	C352	NP_068695.1	actinin alpha 4	10.4	32.9
CSGPGLSPGM*VR	C1453	NP_034357.2	filamin, alpha	1.5	28.2
CSVNLANK	C255	NP_034341.2	four and a half LIM domains 1 isoform 3	25.7	33.9
CSYDEHAK	C58	NP_033784.2	albumin	26.2	36.9
CSYQPTM*EGVHTVHVTFAGVPIPR	C444	NP_034357.2	filamin, alpha	17.3	28.1
CVNLIGK	C1173	NP_032019.2	fibrillin 1	65.7	79.7
CVSTLLDLIQTK	C391	NP_082191.1	adaptor-related protein complex 2, beta 1 subunit isoform b	16.6	25.4
CVVVGDGAVGK	C24	NP_075764.1	ras homolog gene family, member J	53.8	46.9
CYEMASHLR	C128	NP_035202.1	profilin 1	28.9	39.7
CAPGVVGPTEADIDFDIIR	C810	NP_034357.2	filamin, alpha	22.9	36.1
CDIDIR	C333	NP_113581.1	actin-like 6B	20.4	37.9
CIVVEEGK	-	NP_062745.1	destrin	29.3	45.8
CLHPLASETFVSK	C71	NP_034341.2	four and a half LIM domains 1 isoform 3	21.7	32.0
CNEGYEVAPDGR	C1960	NP_032019.2	fibrillin 1	52.8	58.5
CNIHYTGR	-	NP_034859.2	lysyl oxidase-like 1	97.3	96.5
CSNNGLVAGFQSR	-	NP_062733.1	dermatopontin	79.0	73.4
CSPDPGLTALLSDHR	-	NP_059067.2	hemopexin	40.4	66.6
DAFCVFEQNQGLPLR	C430	NP_033805.1	amine oxidase, copper containing 3	48.2	57.6
DGIILCEFINK	C61	NP_034052.2	calponin 1	4.8	31.9
DGSASGTTLLEALDCILPPTRPTDKPLR	C234	NP_034236.2	eukaryotic translation elongation factor 1 alpha 1	7.7	10.8
EDQSILCTGESGAGK	C172	NP_071855.2	myosin, heavy polypeptide 9, non-muscle isoform 1	8.3	18.8
EEPCLLKR	C1989	NP_034357.2	filamin, alpha	6.9	15.7
EIVHIQAGQCGNQIGAK	C12	NP_075768.1	tubulin, beta 3	16.7	30.7
EKLCYVALDFENEM*ATAASSSSLEK	C219	NP_033738.1	actin, alpha, cardiac	46.8	71.3
ELAHSLAELQSCPQEEGPGGR	C408	NP_038898.1	smoothelin	15.3	37.7
EYEELCPR	C1010	NP_032019.2	fibrillin 1	52.3	63.0
EYLPIGGLAEFCK	C106	NP_034455.1	glutamate oxaloacetate transaminase 2, mitochondrial	12.1	16.2
FCTGLTQIETLFK	C254	NP_067248.1	creatine kinase, brain	26.8	23.0
FNAHGDANTIVCNTK	C61	NP_032521.1	lectin, galactose binding, soluble 1	19.6	45.2
FTPAVCGLR	-	NP_001107021.1	latent transforming growth factor beta binding protein 4 isoform b	74.1	79.0
FVFHNEQVYCPDCAK	C273;C276	NP_034341.2	four and a half LIM domains 1 isoform 3	24.5	31.3
GCDVVVIPAGVPR	C93	NP_032643.2	malate dehydrogenase 2, NAD (mitochondrial)	18.9	24.8
GCTFLVGLIQK	C405	NP_033924.2	calpain 2	16.7	35.7
GENHCGIESEIVAGIPR	C319	NP_031824.1	cathepsin B preproprotein	25.5	37.3

GFQFVSSSLPDICYR	-	NP_001103215.1	cellular nucleic acid binding protein isoform 2	56.9	65.1
GHFFVEDQIYCEK	C305	NP_058557.2	carboxyl terminal LIM domain protein 1	20.6	40.3
GIFPVLCK	C474	NP_035229.2	pyruvate kinase, muscle	4.7	17.9
GLGTDEDSLIEIICSR	C133	XP_001474833.1	PREDICTED: similar to Annexin A2 (Annexin II) (Lipocortin II) (Calpactin I heavy chain) (Chromobindin-8) (p36) (Protein I) (Placental anticoagulant protein IV) (PAP-IV)	8.3	14.5
GQCVKPLFGAVTK	C672	NP_032019.2	fibrillin 1	63.9	76.3
GSHCSGSGDPAEYNLR	C572	NP_001002011.2	lamin A isoform A	8.9	26.9
GSLGTSGETCR	C892	NP_032331.2	perlecan (heparan sulfate proteoglycan 2)	39.9	63.8
GTFASLSELHCDK	C94	NP_058652.1	hemoglobin, beta adult minor chain	13.2	20.3
GVVNFAVVITDGHVTGSPCGGIK	C185	NP_666119.1	collagen, type VI, alpha 2	54.4	69.0
HEQNIDCGGGYVK	C105	NP_031617.1	calreticulin	21.5	31.0
HFCPNVPIILVGNK	C107	NP_031510.2	ras homolog gene family, member C	15.4	20.8
HHCPNTPIILVGTK	C105	NP_033033.1	RAS-related C3 botulinum substrate 1	9.3	26.3
HPNSFICLK	C288	NP_032786.1	osteoglycin	29.0	52.9
HSQTTDDPLCPPGTK	-	NP_034061.2	collagen, type IV, alpha 1	22.9	83.8
IASVKPSDAGTYVCQAQNALGTAQK	-	XP_001477365.1	PREDICTED: similar to perlecan (heparan sulfate proteoglycan 2)	56.1	69.4
IDQLECDHQLIQEALIFDNK	C690	NP_598917.1	actinin, alpha 1	10.8	4.1
IIPGFMCQGGDFTR	C62	XP_001002180.2	PREDICTED: hypothetical protein	11.4	32.8
IIYGGSVTGATCK	C218	NP_033441.1	triosephosphate isomerase 1	11.9	39.7
ILYSQCGDVM*R	C32	NP_034990.1	myosin, light polypeptide 6, alkali, smooth muscle and non-muscle	20.7	52.4
IQLEHHISPGDFPDCQK	C356	NP_694708.2	EH-domain containing 2	5.6	19.7
ISPDLCGR		NP_032019.2	fibrillin 1	53.2	71.7
IVSPSGAAVPCK	C1018	NP_034357.2	filamin, alpha	1.6	2.6
IVSPSGAAVPCKVEPGLGADNSVVR	C1018	NP_034357.2	filamin, alpha	5.0	37.0
KITISDCGQL	C161	XP_001002180.2	PREDICTED: hypothetical protein	21.4	40.4
KPVDQYEDCYLAR	C260	NP_598738.1	transferrin	47.3	73.1
KTYITDPVSAPCAPPLQPK	C365	NP_848780.2	LIM domain containing preferred translocation partner in lipoma	8.9	17.6
LCSEQGNHPK	C554	NP_038635.1	myosin, heavy polypeptide 11, smooth muscle	12.7	31.5
LCSGVLGTVVHGK	C79	NP_062732.1	mitochondrial carrier homolog 2	17.0	16.4
LCYVALDFEQEMATAASSSSLEK	C217	NP_031419.1	actin, beta, cytoplasmic	28.8	16.1
LHQVSPVDSGEYVCR	-	XP_001477365.1	PREDICTED: similar to perlecan (heparan sulfate proteoglycan 2)	42.6	38.5

LIENICWTLMDHLGYPK	C1193	NP_001074785.1	dermatan sulfate epimerase-like	98.0	99.0
LLCGLLSDR	C81	XP_001481126.1	PREDICTED: similar to macrophage migration inhibitory factor isoform 2	14.4	31.2
LPCQLHQVIVAR	C640	NP_033924.2	calpain 2	37.1	49.1
LPCVAAK	C211	NP_080720.1	citrate synthase	14.3	23.3
LVVECVM*K	C118	NP_077717.1	fatty acid binding protein 4, adipocyte	22.2	22.9
LVVNFQCDK	C669	NP_033399.1	transglutaminase 2, C polypeptide	11.0	32.0
M*TEEEVEM*LVAGHEDSNGCINYEELVR	C138	NP_034990.1	myosin, light polypeptide 6, alkali, smooth muscle and non-muscle	11.1	20.3
MIPSLCTHGK	-	NP_032019.2	fibrillin 1	74.8	87.9
NFHENCYR	C334	NP_598515.3	filamin binding LIM protein 1	26.7	41.0
NLDSTTVAVHGEEIYCK	C58	NP_031817.1	cysteine and glycine-rich protein 1	18.1	27.3
NPLSDPLYDCIFTVEGAGLTK	C619	NP_033399.1	transglutaminase 2, C polypeptide	1.4	11.3
NTGIICTIGPASR	C49	NP_035229.2	pyruvate kinase, muscle	1.5	5.2
QACILM*IK	C747	NP_038635.1	myosin, heavy polypeptide 11, smooth muscle	9.9	19.0
QIVSSIGLCR		NP_001025007.1	nephronectin isoform b	80.1	84.8
QVQSLTCEVDALK	C328	NP_035831.2	vimentin	4.9	13.8
SEDCFILDHGR	C329	NP_666232.2	gelsolin	9.4	15.8
SIQHCSIR	C119	NP_032019.2	fibrillin 1	79.9	87.8
SPVQCVSPELALTIALNPGGRPK	C1079	XP_619639.3	PREDICTED: tensin 1	2.2	27.9
SQSVRPGADVTFICTAK	C1265	XP_001477365.1	PREDICTED: similar to perlecan (heparan sulfate proteoglycan 2)	58.3	53.7
STLTDSLVCK	C41	NP_031933.1	eukaryotic translation elongation factor 2	11.7	20.7
TDQVCINLR	-	XP_001472266.1	PREDICTED: similar to Epidermal growth factor-containing fibulin-like extracellular matrix protein 1	76.3	83.3
TFCQLILDPIFK	C290	NP_031933.1	eukaryotic translation elongation factor 2	7.2	12.2
TNLLQVCER	C985	NP_033528.3	vinculin	6.2	14.1
TPCEEILVK	C290	NP_034357.2	filamin, alpha	15.4	27.9
TVCAHEELLR	-	NP_032572.1	microfibril-associated glycoprotein 2	80.1	87.5
TVFAEHISDECK	C114	XP_001477314.1	PREDICTED: similar to ribosomal protein L3 isoform 1	6.7	26.6
TVYHAEEVQCDGR	C25	NP_031818.3	cysteine and glycine-rich protein 2	28.2	42.6
VAVPSTIHCDHLIEAQVGGEK	C126	NP_542364.1	aconitase 2, mitochondrial	4.3	15.4
VCHLVGINVTDFTR	C382	NP_038635.1	myosin, heavy polypeptide 11, smooth muscle	17.0	27.1
VCNPIITK	C603	NP_112442.2	heat shock protein 8	3.7	6.3
VEDMAELTCLNEASVLHNLR	C95	NP_038635.1	myosin, heavy polypeptide 11, smooth muscle	5.1	12.7
VGAFTVVCK	C295	NP_034455.1	glutamate oxaloacetate transaminase 2, mitochondrial	14.5	22.7

VGELCAGK	C313	NP_033528.3	vinculin	31.0	40.3
VGINDFCPM*GFGVK	C322	NP_031568.2	biglycan	21.7	74.3
VGTECGNQK	C574	NP_034357.2	filamin, alpha	12.4	27.8
VGVNDFCPTVPK	C326	NP_079987.2	asporin	28.4	36.6
VLHDGGCSLPILR	-	NP_783572.2	latent transforming growth factor beta binding protein 4 isoform a	71.7	74.4
VPFLVLECPNLK	C14	NP_079785.1	DC2 protein	6.4	11.1
VPSGLYLGTCER	C1156	NP_032331.2	perlecan (heparan sulfate proteoglycan 2)	51.4	61.4
VPTPNVSVVDLTCR	C245	XP_001473992.1	PREDICTED: similar to Glyceraldehyde-3-phosphate dehydrogenase (GAPDH) isoform 1	4.4	11.9
VSHALAEGLGVIACIGEK	C127	NP_033441.1	triosephosphate isomerase 1	41.5	40.2
VTYCPTEPGNYIINIK	C2099	NP_034357.2	filamin, alpha	16.0	41.1
VVQCSDLGLDK	C62	NP_031859.1	decorin	34.6	45.8
VVQCSDLGLK	-	NP_031568.2	biglycan	27.5	36.5
YGISLCQAILDETK	C324	NP_034860.2	annexin A1	25.1	52.6
YLIVVTDGHPLEGYKEPC GGLEDAVNEAK	C168	NP_034063.1	collagen, type VI, alpha 1	52.4	72.4
YSGCLTESNLIK	C553	NP_033399.1	transglutaminase 2, C polypeptide	19.2	33.3
YTGHHAYASGCTISPY	-	NP_034858.2	lysyl oxidase	85.1	88.4
YVICVR	C1715	NP_034357.2	filamin, alpha	12.0	34.7
