# Supplementary material for: Disturbed Flow Enhances Inflammatory Signaling and Atherogenesis by Increasing Thioredoxin-1 Level in Endothelial Cell Nuclei
Source: PLoS One. 2014 Sep 29;9(9):e108346. doi: 10.1371/journal.pone.0108346 (PMC4180949; doi:10.1371/journal.pone.0108346)
Supplement: Table S2 — Redox ICAT/MS-measured % oxidation of peptidyl Cys/proteins of RCA and LCA in NLS-Trx1 Tg mice. The data show 193 peptidyl Cys with % oxidation (%ox) value identified from RCA and LCA of Tg mice. (PDF) [file pone.0108346.s002.pdf]

Table S2. Redox ICAT/MS-measured % oxidation of peptidyl Cys/proteins of RCA and LCA in NLS-Trx1 Tg mice. The data show 193 peptidyl Cys with % oxidation (%ox) value identified from RCA and LCA of Tg mice.

Peptide	Cys	Acccession	Annotation	%ox RCA	%ox LCA
ACPVGHHLHR	C864	NP_783572.2	latent transforming growth factor beta binding protein 4 isoform a	60.8	85.2
AEGSDVANAVLDGADCIM*LSGETAK	C358	NP_035229.2	pyruvate kinase, muscle	16.1	23.1
AETFTFHSDICTLPEK	-	NP_033784.2	albumin	73.0	87.8
AGLSSGFVGCVR	C3248	XP_001477365.1	PREDICTED: similar to perlecan (heparan sulfate proteoglycan 2)	39.0	68.2
AHEPTYFTVDCTEAGQGDVSIGIK	C796	NP_034357.2	filamin, alpha	42.8	20.1
AHGQDLGTAGSCLR	C1493	NP_034061.2	collagen, type IV, alpha 1	59.8	75.2
AHNQDLGLAGSCLAR	C1532	NP_034062.2	procollagen, type IV, alpha 2	47.6	77.9
AHSVEECR	C888	XP_001477365.1	PREDICTED: similar to perlecan (heparan sulfate proteoglycan 2)	6.4	5.1
AHVAPCFDASK	C1157	NP_034357.2	filamin, alpha	14.4	22.6
ALANSLACQGK	C339	NP_031464.1	aldolase 1, A isoform	17.4	27.9
ALIQCAK	C950	NP_033528.3	vinculin	20.6	30.0
APSVANIGSHCDLSLK	C2152	NP_034357.2	filamin, alpha	13.9	29.4
ASPFIECHGR	C1615	NP_031760.2	procollagen, type IV, alpha 3	69.6	83.8
AVIFCLSADK	C39	NP_062745.1	destrin	39.4	58.6
AVLFCLSEDK	C39	XP_001480334.1	PREDICTED: similar to Cofilin-1 (Cofilin, non-muscle isoform)	17.9	47.9
CAELEEELK	C190	NP_077745.2	tropomyosin 1, alpha	5.3	33.0
CAPGVVGPTEADIDFDIIR	C810	NP_034357.2	filamin, alpha	1.4	41.7
CATSTPAFFAEK	C270	NP_034860.2	annexin A1	19.4	33.8
CAVVDVPFGGAK	C172	NP_032159.1	glutamate dehydrogenase 1	16.4	26.2
CDIDIR	C333	NP_113581.1	actin-like 6B	21.5	43.9
CDKEVYFAER	C7	NP_031789.1	cysteine-rich protein 1 (intestinal)	37.9	55.8
CDLEIQANGR	C10	NP_033399.1	transglutaminase 2, C polypeptide	8.3	6.1
CDRVDQLTAQLADLAAR	C545	NP_033528.3	vinculin	28.5	83.4
CDSGFALDSEER	C1057	NP_032019.2	fibrillin 1	51.4	79.6
CDVDIR	C286	NP_780706.1	actin, beta-like 2	21.4	45.5
CGSGIVGAVVK	C249	NP_058078.1	PDZ and LIM domain 3	28.8	51.4
CGTGIVGVFVK	C261	NP_058557.2	carboxyl terminal LIM domain protein 1	36.8	52.9
CIESLIAVFQK	C8	NP_058020.1	S100 calcium binding protein A11 (calizzarin)	3.1	15.4
CIIPNHEKR	C678	NP_780469.1	myosin heavy chain 10, non-muscle	3.2	13.4
CILLSNLSNTSHAPK	C1464	NP_032014.3	fatty acid synthase	11.3	31.6
CLDAFPNLR	C174	NP_034489.1	glutathione S-transferase, mu 3	14.3	18.9

CLELFSELAEDK	C412	NP_032328.2	heat shock protein 1, beta	63.3	26.9
CLGIPTR	C290	NP_033399.1	transglutaminase 2, C polypeptide	12.5	12.3
CLHPLASETFVSK	C71	NP_034341.2	four and a half LIM domains 1 isoform 3	19.9	45.1
CLIEILASR	C111	NP_036052.2	annexin A10	4.5	0.9
CNGVLEGIR	C694	NP_071855.2	myosin, heavy polypeptide 9, non-muscle isoform 1	22.6	37.7
CPETLFQPSFIGMESAGIHETTYNSIMK	C259	NP_033738.1	actin, alpha, cardiac	12.3	26.8
CQLEINFNTLQTK	C352	NP_068695.1	actinin alpha 4	9.8	35.0
CSGPGLER	C1165	NP_034357.2	filamin, alpha	15.7	42.0
CSGPGLSPGM*VR	C1453	NP_034357.2	filamin, alpha	17.1	29.1
CSLCGEESFGTGSDHLR	C340;C343	XP_001477201.1	PREDICTED: hypothetical protein	5.9	35.3
CSPDPGLTALLSDHR	C255	NP_059067.2	hemopexin	45.0	71.6
CSQAVYAAEK	C122	NP_031817.1	cysteine and glycine-rich protein 1	37.3	59.8
CSVNLANK	C255	NP_034341.2	four and a half LIM domains 1 isoform 3	25.1	44.9
CSYDEHAK	C58	NP_033784.2	albumin	25.1	47.3
CSYQPTMEGVHTVHVTFAGVPIPR	C444	NP_034357.2	filamin, alpha	15.1	29.3
CVSTLLDLIQTK	C391	NP_082191.1	adaptor-related protein complex 2, beta 1 subunit isoform b	9.6	27.3
CAPGVVGPTEADIDFDIIR	C810	NP_034357.2	filamin, alpha	10.9	41.7
CECFPGLAVGLDGR	C639;C641	NP_032019.2	fibrillin 1	52.4	76.7
CFELQALLEEER	C516	NP_114397.3	sarcolemma associated protein	17.9	18.7
CGEVVQEHVIR	C248	NP_598515.3	filamin binding LIM protein 1	32.5	62.5
CIVVEEGK		NP_062745.1	destrin	26.3	44.3
CNEGYEVAPDGR	C1960	NP_032019.2	fibrillin 1	45.1	83.7
CNIHYTGR		NP_034859.2	lysyl oxidase-like 1	97.5	93.9
CQCHLR	C53;C55	NP_031859.1	decorin	22.9	64.0
CYEM*ASHLR	C128	NP_035202.1	profilin 1	22.2	36.4
DAFCVFEQNQGLPLR	C430	NP_033805.1	amine oxidase, copper containing 3	42.3	57.0
DFASCHLAQAPNHVVVSR	-	NP_598738.1	transferrin	45.7	79.0
DGIILCEFINK	C61	NP_034052.2	calponin 1	1.1	28.6
DGLGFCALIHR	C180	NP_598917.1	actinin, alpha 1	15.1	21.2
DGSASGTTLLEALDCILPPTRPTDKPLR	C234	NP_034236.2	eukaryotic translation elongation factor 1 alpha 1	14.1	19.7
DICNDVLSLLEK	C94	XP_001477753.1	PREDICTED: similar to 14-3-3 zeta	28.8	36.4
DLAGCIHGLSNVK	C418	NP_766599.1	isocitrate dehydrogenase 2 (NADP+), mitochondrial	21.6	23.6
DQGPNVCALQQILGTK	C46	NP_056599.1	periostin, osteoblast specific factor	16.6	52.6
DSNNLCLHFNPR	C43	NP_032521.1	lectin, galactose binding, soluble 1	22.7	44.7
DVPLGAPLCIIVEK	C290	NP_663589.2	dihydrolipoamide S-acetyltransferase (E2 component of pyruvate	7.4	36.0

			dehydrogenase complex)		
EDQSILCTGESGAGK	C172	NP_071855.2	myosin, heavy polypeptide 9, non-muscle isoform 1	5.5	23.3
EEIVYLPCIYR	C31	NP_033176.2	selenium binding protein 1	12.0	34.8
EFNGLGDCLTK	C160	NP_031476.3	solute carrier family 25 (mitochondrial carrier, adenine nucleotide translocator), member 4	9.5	16.0
EIVHIQAGQCGNQIGAK	C12	NP_075768.1	tubulin, beta 3	14.7	23.6
EKPYFPIPEDCTFIQNVPLEDR	C449	NP_058085.2	heterogeneous nuclear ribonucleoprotein U	14.2	23.4
ELETVCNDVLALLDK	C97	NP_035868.1	tyrosine 3-monooxygenase/tryptophan 5-monooxygenase activation protein, eta polypeptide	37.1	32.6
ELGSPPGISLETIDAAFSCPGSSR	C364	NP_059067.2	hemopexin	45.6	59.6
EPCGGLEDAVNEAK	C168	NP_034063.1	collagen, type VI, alpha 1	58.2	89.7
ESNPALGIDCLHK	C493	NP_031663.1	chaperonin subunit 5 (epsilon)	16.6	30.0
EVESVTPEHCIFASNTSALPINQIAAVSK	C470	NP_849209.1	mitochondrial trifunctional protein, alpha subunit	9.5	23.5
EYEELCPR	C1010	NP_032019.2	fibrillin 1	50.0	77.8
EYLPIGGLAEFCK	C106	NP_034455.1	glutamate oxaloacetate transaminase 2, mitochondrial	8.1	10.9
FCTGLTQIETLFK	C254	NP_067248.1	creatine kinase, brain	4.8	9.9
FEELCSDLFR	C306	NP_034608.2	heat shock 70kDa protein 1B	17.6	37.1
FGEVVDCTIK	C104	NP_001041526.1	heterogeneous nuclear ribonucleoprotein A/B isoform 1	29.3	23.6
FM*TVLCTR	C546	NP_001103681.1	annexin A6 isoform b	21.0	39.3
FNAHGDANTIVCNTK	C61	NP_032521.1	lectin, galactose binding, soluble 1	19.7	42.1
FNAILCSR	C180	XP_001473013.1	PREDICTED: similar to Annexin A11	16.1	27.9
FTFEEAEAECTSR	C270	NP_001074718.1	versican	43.9	66.9
FTPAVCGLR	-	NP_001107021.1	latent transforming growth factor beta binding protein 4 isoform b	74.5	90.3
FVFHNEQVYCPDCAK	C273;C276	NP_034341.2	four and a half LIM domains 1 isoform 3	23.1	43.7
GAGTDEGCLIEILASR	C108	NP_038499.2	annexin A4	24.8	20.0
GAPLVVICQGK	C448	NP_033494.1	dihydropyrimidinase-like 3	17.2	34.8
GCDVVVIPAGVPR	C93	NP_032643.2	malate dehydrogenase 2, NAD (mitochondrial)	13.1	30.5
GCTFLVGLIQK	C405	NP_033924.2	calpain 2	16.6	17.4
GEDFYCVTCHETK	C150;C153	NP_034341.2	four and a half LIM domains 1 isoform 3	19.7	56.0
GFGTDEQAIIDCLGSR	C224	NP_038497.2	annexin A11	22.0	27.7
GFQFVSSSLPDICYR	-	NP_001103215.1	cellular nucleic acid binding protein isoform 2	51.2	69.3
GHFFVEDQIYCEK	C305	NP_058557.2	carboxyl terminal LIM domain protein 1	24.2	32.9
GIFPVLCK	C474	NP_035229.2	pyruvate kinase, muscle	5.5	3.7

GLGTDEDSLIEIICSR	C133	XP_001474833.1	PREDICTED: similar to Annexin A2 (Annexin II) (Lipocortin II) (Calpactin I heavy chain) (Chromobindin-8) (p36) (Protein I) (Placental anticoagulant protein IV) (PAP-IV)	9.2	28.0
GQCVKPLFGAVTK	C672	NP_032019.2	fibrillin 1	66.4	87.4
GSHCSGSGDPAEYNLR	C572	NP_001002011.2	lamin A isoform A	4.7	26.7
GSLGTSGETCR	C892	NP_032331.2	perlecan (heparan sulfate proteoglycan 2)	24.0	69.1
GTFASLSELHCDK	C94	NP_058652.1	hemoglobin, beta adult minor chain	11.9	26.4
GVLFGVPGAFTPGCSK	C96	NP_036151.1	peroxiredoxin 5 precursor	18.0	33.8
GVVNFAVVITDGHVTGSPCGGIK	C185	NP_666119.1	collagen, type VI, alpha 2	49.2	70.7
HDLDLICR	C245	NP_114074.1	protein phosphatase 1, catalytic subunit, alpha	10.2	23.9
HEQNIDCGGGYVK	C105	NP_031617.1	calreticulin	26.6	27.7
HFCPNVPIILVGNK	C107	NP_031510.2	ras homolog gene family, member C	16.0	15.3
HGCTVLTALGTILK	C67	NP_038621.2	myoglobin	18.9	20.8
HHCPNTPIILVGTK	C105	NP_033033.1	RAS-related C3 botulinum substrate 1	5.6	8.6
HPNSFICLK	C288	NP_032786.1	osteoglycin	30.1	47.3
HSQTTDDPLCPPGTK		NP_034061.2	collagen, type IV, alpha 1	21.6	29.7
IDQLECDHQLIQEALIFDNK	C690	NP_598917.1	actinin, alpha 1	25.5	10.0
IIPGFMCQGGDFTR	C62	XP_001002180.2	PREDICTED: hypothetical protein	10.5	22.3
ILYSQCGDVM*R	C32	NP_034990.1	myosin, light polypeptide 6, alkali, smooth muscle and non-muscle	10.3	35.6
IQLEHHISPGDFPDCQK	C356	NP_694708.2	EH-domain containing 2	4.6	10.1
ISLGLPVGAVINCADNTGAK	C28	NP_075029.1	ribosomal protein L23	12.7	22.8
IVSPSGAAVPCK	C1018	NP_034357.2	filamin, alpha	9.0	19.6
KITISDCGQL	C161	XP_001002180.2	PREDICTED: hypothetical protein	18.1	41.2
KPVDQYEDCYLAR	C260	NP_598738.1	transferrin	54.9	78.4
KQELEEICHDLEAR	C917	NP_071855.2	myosin, heavy polypeptide 9, non-muscle isoform 1	16.9	31.5
KTYITDPVSAPCAPPLQPK	C365	NP_848780.2	LIM domain containing preferred translocation partner in lipoma	8.8	16.1
LCYVALDFENEMATAASSSSLEK	C219	NP_033738.1	actin, alpha, cardiac	13.4	37.3
LFACSNR	C670	NP_666232.2	gelsolin	14.7	26.9
LFDSICNNK	C106	XP_001475135.1	PREDICTED: similar to Guanine nucleotide-binding protein G(i), alpha-2 subunit (Adenylate cyclase-inhibiting G alpha protein)	18.6	21.7
LFQEEFPGIPYPPDAAVECHR	C148	NP_059067.2	hemopexin	22.2	68.4
LLCGLLSDR	C81	XP_001481126.1	PREDICTED: similar to macrophage migration inhibitory factor isoform 2	11.6	41.7

LPAIEPSDQGQYLCR	C1405	XP_001477365.1	PREDICTED: similar to perlecan (heparan sulfate proteoglycan 2)	51.5	74.9
LPCVAAK	C211	NP_080720.1	citrate synthase	17.6	24.5
LPCVEDYLSAILNR	-	NP_033784.2	albumin	82.9	94.6
LPVVIGGLLDVDCSEDVIK	C824	NP_001003908.1	clathrin, heavy polypeptide (Hc)	14.6	20.1
LQVEPAVDTSGVQCYGPGIEGQGVFR	C1260	NP_034357.2	filamin, alpha	12.2	22.5
LTLVCSTAPGPLELDLTGDLESFKK	C79	NP_598557.3	Rho GDP dissociation inhibitor (GDI) alpha	17.3	30.0
LTTPTYGDLNHLVSATMSGVTTCLR	C238	XP_001473173.1	PREDICTED: similar to Tubulin, beta 4	17.0	25.5
LVQCVDAFEEK	C1555	NP_647461.3	myosin, light polypeptide kinase	20.8	29.4
LVVECVM*K	C118	NP_077717.1	fatty acid binding protein 4, adipocyte	20.2	42.1
LVVNFQCDK	C669	NP_033399.1	transglutaminase 2, C polypeptide	15.5	31.8
M*IPSLCTHGK	C1041	NP_032019.2	fibrillin 1	97.7	99.3
NECFLQHK	C125	NP_033784.2	albumin	73.5	83.3
NECLEAGALFQDPSFPALPSSLGYK	C39	NP_033924.2	calpain 2	19.8	20.8
NLDSTTVAIHDEEIYCK	C58	NP_031818.3	cysteine and glycine-rich protein 2	26.7	49.9
NLDSTTVAVHGEEIYCK	C58	NP_031817.1	cysteine and glycine-rich protein 1	8.2	44.1
NPLSDPLYDCIFTVEGAGLTK	C619	NP_033399.1	transglutaminase 2, C polypeptide	13.1	1.3
NSFTVDCSK	C2661	NP_001074654.1	filamin C, gamma	6.1	30.8
NTGIICTIGPASR	C49	NP_035229.2	pyruvate kinase, muscle	5.7	11.5
QIVSSIGLCR		NP_001025007.1	nephronectin isoform b	75.4	93.3
QVQSLTCEVDALK	C328	NP_035831.2	vimentin	3.4	20.2
SEDCFILDHGR	C329	NP_666232.2	gelsolin	9.7	28.1
SFLIEIQCISR	C143	NP_031642.1	caveolin, caveolae protein 1	19.7	26.1
SGNCYLDIRPR	C1536	NP_032019.2	fibrillin 1	52.0	88.5
SICTTVLELLDK	C136	XP_001473550.1	PREDICTED: similar to Ywhaq protein	31.9	40.3
SKEEAFDAICQLIAGK	C100	NP_080757.1	tubulin polymerization-promoting protein family member 3	23.0	33.2
SPVQCVSPELALTIALNPGGRPK	C1079	XP_619639.3	PREDICTED: tensin 1	13.7	25.1
SQSVRPGADVTFICTAK	C1265	XP_001477365.1	PREDICTED: similar to perlecan (heparan sulfate proteoglycan 2)	46.0	86.1
SSCISQHVISEAK	C395	NP_783572.2	latent transforming growth factor beta binding protein 4 isoform a	50.5	74.9
STAISCIGAYR	-	NP_001035516.1	thrombospondin, type I, domain containing 4 isoform a	86.8	94.5
STLTDSLVCK	C41	NP_031933.1	eukaryotic translation elongation factor 2	10.2	33.5
TCDLLASFK	C102	NP_033280.1	serine (or cysteine) proteinase inhibitor, clade B, member 6a	18.1	25.3

TDQVCINLR	-	XP_001472266.1	PREDICTED: similar to Epidermal growth factor-containing fibulin-like extracellular matrix protein 1	71.8	97.9
TECGLLGFVTDLK	C538	NP_998781.1	plasma membrane calcium ATPase 4	16.8	21.0
TFCQLILDPIFK	C290	NP_031933.1	eukaryotic translation elongation factor 2	4.6	22.4
TFYSCTTEGR	-	NP_034363.1	fibronectin 1	73.1	89.8
TICIETIK	C847	NP_032019.2	fibrillin 1	53.1	83.2
TIIPLISQCTPK	C212	NP_032643.2	malate dehydrogenase 2, NAD (mitochondrial)	11.4	34.2
TLTGTVIDSGDGVTHVIPVAEGYVIGSCIK	C189	NP_076224.1	ARP3 actin-related protein 3 homolog	17.7	25.2
TNLLQVCER	C985	NP_033528.3	vinculin	16.0	19.7
TPCEEILVK	C2593	NP_034357.2	filamin, alpha	5.6	0.8
TVYFAEEVQCEGNSFHK	C25	NP_031817.1	cysteine and glycine-rich protein 1	26.8	51.3
TVYHAEEVQCDGR	C25	NP_031818.3	cysteine and glycine-rich protein 2	31.3	56.9
VACITEQVLTLVNK	C478	NP_598694.2	ribophorin I	10.6	15.3
VCNPIITK	C603	NP_112442.2	heat shock protein 8	10.1	6.4
VCRTWLYNLK	C1217	XP_001481380.1	PREDICTED: doublecortin domain containing 5	6.2	2.6
VDINTEDLEDGTCR	C2094	NP_034357.2	filamin, alpha	26.3	49.8
VGEATETALTCLVEK	C447	NP_001103610.1	ATPase, Ca++ transporting, cardiac muscle, slow twitch 2 isoform a	15.3	25.5
VGINDFCPMGFGVK	C322	NP_031568.2	biglycan	40.9	45.7
VGTECGNQK	C574	NP_034357.2	filamin, alpha	12.4	31.8
VGVNDFCPTVPK	C326	NP_079987.2	asporin	17.7	51.6
VHSPSGALEECYVTEIDQDK	C2370	NP_034357.2	filamin, alpha	27.8	53.7
VIHLQFNSISSLTDDTFCK	C255	NP_032786.1	osteoglycin	18.9	55.6
VLHDGGCSLPILR	-	NP_783572.2	latent transforming growth factor beta binding protein 4 isoform a	62.6	96.4
VPFLVLECPNLK	C14	NP_079785.1	DC2 protein	9.6	15.8
VPSGLYLGTCER	C1156	NP_032331.2	perlecan (heparan sulfate proteoglycan 2)	54.3	89.9
VPTPNVSVVDLTCR	C245	XP_001473992.1	PREDICTED: similar to Glyceraldehyde-3-phosphate dehydrogenase (GAPDH) isoform 1	14.3	25.9
VQELGHGCSALVTK	C1927	NP_035732.1	talin 1	18.2	28.6
VQVQDNEGCSVEATVK		NP_034357.2	filamin, alpha	16.3	94.7
VSCLEIPGPHGPK		NP_666119.1	collagen, type VI, alpha 2	70.0	84.6
VSHALAEGLGVIACIGEK	C127	NP_033441.1	triosephosphate isomerase 1	10.1	37.8
VTYCPTEPGNYIINIK	C2099	NP_034357.2	filamin, alpha	11.1	61.9
VVQCSDLGLDK	C62	NP_031859.1	decorin	31.3	53.9
VVQCSDLGLK	C77	NP_031568.2	biglycan	13.1	49.3
YFLVGAGAIGCELLK	C539	XP_001474649.1	PREDICTED: similar to ubiquitin activating enzyme E1	9.0	27.6

YGISLCQAILDETK	C324	NP_034860.2	annexin A1	24.3	21.1
YSGCLTESNLIK	C553	NP_033399.1	transglutaminase 2, C polypeptide	21.3	32.3
YTGHHAYASGCTISPY	-	NP_034858.2	lysyl oxidase	72.7	89.1
YVICVR	C1715	NP_034357.2	filamin, alpha	26.1	24.3
