# Supplementary material for: Disturbed Flow Enhances Inflammatory Signaling and Atherogenesis by Increasing Thioredoxin-1 Level in Endothelial Cell Nuclei
Source: PLoS One. 2014 Sep 29;9(9):e108346. doi: 10.1371/journal.pone.0108346 (PMC4180949; doi:10.1371/journal.pone.0108346)
Supplement: Table S3 — Redox ICAT/MS-measured % oxidation of peptidyl Cys/proteins of LCA in WT and Tg mice. The data show 156 peptidyl Cys with % oxidation (%ox) value identified from LCA of WT and Tg mice. (PDF) [file pone.0108346.s003.pdf]

Table S3. Redox ICAT/MS-measured % oxidation of peptidyl Cys/proteins of LCA in WT and Tg mice. The data show 156 peptidyl Cys with % oxidation (%ox) value identified from LCA of WT and Tg mice.

Peptide	Cys	Accession	Annotation	%ox WT	%ox Tg
ACPVGHHLHR	C864	NP_783572.2	latent transforming growth factor beta binding protein 4 isoform a	67.6	85.2
AGLSSGFVGCVR	C3248	XP_001477365.1	PREDICTED: similar to perlecan (heparan sulfate proteoglycan 2)	46.8	68.2
AHGQDLGTAGSCLR	C1493	NP_034061.2	collagen, type IV, alpha 1	75.1	75.2
AHNQDLGLAGSCLAR	C1532	NP_034062.2	procollagen, type IV, alpha 2	67.1	86.0
AHSVEECR	C888	XP_001477365.1	PREDICTED: similar to perlecan (heparan sulfate proteoglycan 2)	88.5	5.1
AHVAPCFDASK	C1157	NP_034357.2	filamin, alpha	29.2	22.6
ALANSLACQGK	C339	NP_031464.1	aldolase 1, A isoform	23.9	27.9
ALEVEECR	C1530	NP_032331.2	perlecan (heparan sulfate proteoglycan 2)	45.3	76.6
ALIQCAK	C950	NP_033528.3	vinculin	32.0	30.0
APSVANIGSHCDLSLK	C2152	NP_034357.2	filamin, alpha	26.7	29.4
ASPFIECHGR	C1615	NP_031760.2	procollagen, type IV, alpha 3	82.8	83.8
AVIFCLSADK	C39	NP_062745.1	destrin	58.5	58.6
CAELEEELK	C190	NP_077745.2	tropomyosin 1, alpha	7.6	33.0
CAPGVVGPTEADIDFDIIR	C810	NP_034357.2	filamin, alpha	40.4	41.7
CATSTPAFFAEK	C270	NP_034860.2	annexin A1	32.4	33.8
CAVVDVPFGGAK	C172	NP_032159.1	glutamate dehydrogenase 1	35.3	26.2
CDIDIR	C333	NP_113581.1	actin-like 6B	37.9	43.9
CDKEVYFAER	C7	NP_031789.1	cysteine-rich protein 1 (intestinal)	42.4	55.8
CDRVDQLTAQLADLAAR	C545	NP_033528.3	vinculin	32.1	83.4
CDSGFALDSEER	C1057	NP_032019.2	fibrillin 1	68.1	79.6
CDVDIR	C286	NP_780706.1	actin, beta-like 2	40.5	45.5
CEKPFLGHR	C227	NP_659111.1	LIM and senescent cell antigen like domains 2	38.9	47.0
CGTGIVGVFVK	C261	NP_058557.2	carboxyl terminal LIM domain protein 1	29.5	52.9
CIESLIAVFQK	C8	NP_058020.1	S100 calcium binding protein A11 (calizzarin)	12.0	15.4
CIIPNHEK	C671	NP_071855.2	myosin, heavy polypeptide 9, non-muscle isoform 1	0.5	18.8
CIIPNHEKR	C678	NP_780469.1	myosin heavy chain 10, non-muscle	18.3	13.4
CILLSNLSNTSHAPK	C1464	NP_032014.3	fatty acid synthase	27.0	31.6
CLGIPTR	C290	NP_033399.1	transglutaminase 2, C polypeptide	9.1	12.3
CLHPLASETFVSK	C71	NP_034341.2	four and a half LIM domains 1 isoform 3	32.0	45.1
CNGVLEGIR	C694	NP_071855.2	myosin, heavy polypeptide 9, non-muscle isoform 1	30.0	37.7
CQLEINFNTLQTK	C352	NP_068695.1	actinin alpha 4	32.9	35.0
CSGPGLSPGM*VR	C1453	NP_034357.2	filamin, alpha	28.2	29.1
CSPDPGLTALLSDHR	C255	NP_059067.2	hemopexin	66.6	71.6
CSVNLANK	C255	NP_034341.2	four and a half LIM domains 1 isoform 3	33.9	44.9
CSYDEHAK	C58	NP_033784.2	albumin	36.9	47.3

CSYQPTM*EGVHTVHVTFAGVPIPR	C444	NP_034357.2	filamin, alpha	28.1	23.9
CVNLIGK	C1173	NP_032019.2	fibrillin 1	79.7	89.5
CVSTLLDLIQTK	C391	NP_082191.1	adaptor-related protein complex 2, beta 1 subunit isoform b	25.4	27.3
CAPGVVGPTEADIDFDIIR	C810	NP_034357.2	filamin, alpha	36.1	41.7
CFELQALLEEER	C516	NP_114397.3	sarcolemma associated protein	36.4	18.7
CGDSVYAAEK	C122	NP_031818.3	cysteine and glycine-rich protein 2	28.8	40.6
CGEVVQEHVIR	C248	NP_598515.3	filamin binding LIM protein 1	48.1	62.5
CIVVEEGK	-	NP_062745.1	destrin	45.8	44.3
CKPGFFGDATK	-	NP_032331.2	perlecan (heparan sulfate proteoglycan 2)	65.7	93.9
CLHPLASETFVSK	C71	NP_034341.2	four and a half LIM domains 1 isoform 3	32.0	45.1
CLIEILASR	C111	NP_036052.2	annexin A10	25.4	20.8
CNEGYEVAPDGR	C1960	NP_032019.2	fibrillin 1	58.5	83.7
CNIHYTGR	-	NP_034859.2	lysyl oxidase-like 1	96.5	93.9
CPETLFQPSFIGM*ESAGIHETTYNSIM*K	C259	NP_033738.1	actin, alpha, cardiac	34.0	30.5
CSNNGLVAGFQSR	-	NP_062733.1	dermatopontin	73.4	90.3
CYEM*ASHLR	C128	NP_035202.1	profilin 1	39.7	36.4
DAFCVFEQNQGLPLR	C430	NP_033805.1	amine oxidase, copper containing 3	57.6	57.0
DFHVHCYR	C567	NP_848780.2	LIM domain containing preferred translocation partner in lipoma	33.2	45.6
DGIILCEFINK	C61	NP_034052.2	calponin 1	31.9	28.6
DGLGFCALIHR	C180	NP_598917.1	actinin, alpha 1	25.5	21.2
DGSASGTTLLEALDCILPPTRPTDKPLR	C234	NP_034236.2	eukaryotic translation elongation factor 1 alpha 1	10.8	19.7
DICNDVLSLLEK	C94	XP_001477753.1	PREDICTED: similar to 14-3-3 zeta	32.1	36.4
DQGPNVCALQQILGTK	C46	NP_056599.1	periostin, osteoblast specific factor	89.4	52.6
DSNNLCLHFNPR	C43	NP_032521.1	lectin, galactose binding, soluble 1	39.3	44.7
EDQSILCTGESGAGK	C172	NP_071855.2	myosin, heavy polypeptide 9, non-muscle isoform 1	18.8	23.3
EIVHIQAGQCGNQIGAK	C12	NP_075768.1	tubulin, beta 3	30.7	23.6
ELAHSLAELQSCPQEEGPGGR	C408	NP_038898.1	smoothelin	37.7	38.6
ELETVCNDVLALLDK	C97	NP_035868.1	tyrosine 3-monooxygenase/tryptophan 5-monooxygenase activation protein, eta polypeptide	27.2	32.6
EYEELCPR	C1010	NP_032019.2	fibrillin 1	63.0	77.8
EYLPIGGLAEFCK	C106	NP_034455.1	glutamate oxaloacetate transaminase 2, mitochondrial	16.2	10.9
FCTGLTQIETLFK	C254	NP_067248.1	creatine kinase, brain	23.0	14.4
FEELCSDLFR	C306	NP_034608.2	heat shock 70kDa protein 1B	37.6	37.1
FNAHGDANTIVCNTK	C61	NP_032521.1	lectin, galactose binding, soluble 1	45.2	42.1
FNAILCSR	C180	XP_001473013.1	PREDICTED: similar to Annexin A11	42.5	27.9
FSSGITGCIK	-	XP_001477365.1	PREDICTED: similar to perlecan (heparan sulfate proteoglycan 2)	63.6	59.2
FTPAVCGLR	-	NP_001107021.1	latent transforming growth factor beta binding protein 4 isoform b	79.0	90.3
FVFHNEQVYCPDCAK	C273;C	NP_034341.2	four and a half LIM domains 1 isoform 3	31.3	46.5

	276				
GCDVVVIPAGVPR	C93	NP_032643.2	malate dehydrogenase 2, NAD (mitochondrial)	24.8	30.5
GCTFLVGLIQK	C405	NP_033924.2	calpain 2	35.7	33.6
GFQFVSSSLPDICYR	-	NP_001103215.1	cellular nucleic acid binding protein isoform 2	65.1	69.3
GHFFVEDQIYCEK	C305	NP_058557.2	carboxyl terminal LIM domain protein 1	40.3	32.9
GIFPVLCK	C474	NP_035229.2	pyruvate kinase, muscle	17.9	3.7
GLGTDEDSLIEIICSR	C133	XP_001474833.1	PREDICTED: similar to Annexin A2 (Annexin II) (Lipocortin II) (Calpactin I heavy chain) (Chromobindin-8) (p36) (Protein I) (Placental anticoagulant protein IV) (PAP-IV)	14.5	30.7
GQCVKPLFGAVTK	C672	NP_032019.2	fibrillin 1	76.3	87.4
GSHCSGSGDPAEYNLR	C572	NP_001002011.2	lamin A isoform A	26.9	26.7
GSLGTSGETCR	C892	NP_032331.2	perlecan (heparan sulfate proteoglycan 2)	63.8	69.1
GTFASLSELHCDK	C94	NP_058652.1	hemoglobin, beta adult minor chain	20.3	26.4
GVVNFAVVITDGHVTGSPCGGIK	C185	NP_666119.1	collagen, type VI, alpha 2	69.0	85.7
HEQNIDCGGGYVK	C105	NP_031617.1	calreticulin	31.0	27.7
HFCPNVPIILVGNK	C107	NP_031510.2	ras homolog gene family, member C	20.8	15.3
HPNSFICLK	C288	NP_032786.1	osteoglycin	52.9	47.3
HSQTTDDPLCPPGTK	-	NP_034061.2	collagen, type IV, alpha 1	83.8	69.6
IASVKPSDAGTYVCQAQNALGTAQK	-	XP_001477365.1	PREDICTED: similar to perlecan (heparan sulfate proteoglycan 2)	69.4	78.1
IDQLECDHQLIQEALIFDNK	C690	NP_598917.1	actinin, alpha 1	4.1	10.0
IIPGFMCQGGDFTR	C62	XP_001002180.2	PREDICTED: hypothetical protein	32.8	22.3
ILYSQCGDVM*R	C32	NP_034990.1	myosin, light polypeptide 6, alkali, smooth muscle and non-muscle	52.4	35.6
IQLEHHISPGDFPDCQK	C356	NP_694708.2	EH-domain containing 2	19.7	10.1
IVSPSGAAVPCK	C1018	NP_034357.2	filamin, alpha	2.6	19.6
KITISDCGQL	C161	XP_001002180.2	PREDICTED: hypothetical protein	40.4	41.2
KPVDQYEDCYLAR	C260	NP_598738.1	transferrin	73.1	78.4
KTYITDPVSAPCAPPLQPK	C365	NP_848780.2	LIM domain containing preferred translocation partner in lipoma	17.6	16.1
LCSGVLGTVVHGK	C79	NP_062732.1	mitochondrial carrier homolog 2	16.4	25.2
LCYVALDFENEMATAASSSSLEK	C219	NP_033738.1	actin, alpha, cardiac	14.7	37.3
LFACSNR	C670	NP_666232.2	gelsolin	7.9	26.9
LFDSICNNK	C106	XP_001475135.1	PREDICTED: similar to Guanine nucleotide-binding protein G(i), alpha-2 subunit (Adenylate cyclase-inhibiting G alpha protein)	36.3	21.7
LHQVSPVDSGEYVCR	-	XP_001477365.1	PREDICTED: similar to perlecan (heparan sulfate proteoglycan 2)	38.5	71.6
LIENICWTLM*DHLGYPK	C1193	NP_001074785.1	dermatan sulfate epimerase-like	99.0	98.4
LLCGLLSDR	C81	XP_001481126.1	PREDICTED: similar to macrophage migration inhibitory factor isoform 2	31.2	41.7
LPCQLHQVIVAR	C640	NP_033924.2	calpain 2	49.1	27.9

LPCVAAK	C211	NP_080720.1	citrate synthase	23.3	24.5
LPCVEDYLSAILNR	-	NP_033784.2	albumin	91.1	94.6
LPVVIGGLLDVDCSED VIK	C824	NP_00100390 8.1	clathrin, heavy polypeptide (Hc)	12.4	20.1
LVVECVM*K	C824	NP_077717.1	fatty acid binding protein 4, adipocyte	22.9	42.1
LVVNFQCDK	C669	NP_033399.1	transglutaminase 2, C polypeptide	32.0	31.8
M*IPSLCTHGK	C1041	NP_032019.2	fibrillin 1	98.4	99.3
NCAAYLK	C836	NP_082297.1	myosin, heavy polypeptide 14	26.7	25.5
NECFLQHK	C125	NP_033784.2	albumin	88.4	83.3
NFHENCYR	C334	NP_598515.3	filamin binding LIM protein 1	41.0	58.7
NIGASVEFHCAVPNER	C2871	XP_00147736 5.1	PREDICTED: similar to perlecan (heparan sulfate proteoglycan 2)	47.7	65.5
NLDSTTVAVHGEEIYC K	C58	NP_031817.1	cysteine and glycine-rich protein 1	27.3	44.1
NPLSDPLYDCIFTVEG AGLTK	C619	NP_033399.1	transglutaminase 2, C polypeptide	11.3	24.7
NTGIICTIGPASR	C49	NP_035229.2	pyruvate kinase, muscle	5.2	11.5
QACILM*IK	C747	NP_038635.1	myosin, heavy polypeptide 11, smooth muscle	19.0	15.0
QIVSSIGLCR	-	NP_00102500 7.1	nephronectin isoform b	84.8	93.3
QVQSLTCEVDALK	C328	NP_035831.2	vimentin	13.8	20.2
SEDCFILDHGR	C329	NP_666232.2	gelsolin	15.8	28.1
SICTTVLELLDK	C136	XP_00147355 0.1	PREDICTED: similar to Ywhaq protein	44.3	40.3
SPVQCVSPELALTIAL NPGGRPK	C1079	XP_619639.3	PREDICTED: tensin 1	27.9	25.1
SQSVRPGADVTFICTA K	C1265	XP_00147736 5.1	PREDICTED: similar to perlecan (heparan sulfate proteoglycan 2)	53.7	75.8
SSCISQHVISEAK	C395	NP_783572.2	latent transforming growth factor beta binding protein 4 isoform a	62.9	89.0
STLTDSLVCK	C41	NP_031933.1	eukaryotic translation elongation factor 2	20.7	33.5
TDQVCINLR	-	XP_00147226 6.1	PREDICTED: similar to Epidermal growth factor-containing fibulin-like extracellular matrix protein 1	83.3	97.9
TFCQLILDPIFK	C290	NP_031933.1	eukaryotic translation elongation factor 2	12.2	22.4
TLTGTVIDSGDGVTHV IPVAEGYVIGSCIK	-	NP_076224.1	ARP3 actin-related protein 3 homolog	20.3	25.2
TNLLQVCER	C985	NP_033528.3	vinculin	14.1	19.7
TPCEEILVK	C290	NP_034357.2	filamin, alpha	0.8	0.8
TVCAHEELLR	-	NP_032572.1	microfibril-associated glycoprotein 2	87.5	96.9
TVFAEHISDECK	C114	XP_00147731 4.1	PREDICTED: similar to ribosomal protein L3 isoform 1	26.6	18.8
TVYHAEEVQCDGR	C25	NP_031818.3	cysteine and glycine-rich protein 2	42.6	56.9
VCHLVGINVTDFTR	C382	NP_038635.1	myosin, heavy polypeptide 11, smooth muscle	27.1	25.1
VCNPIITK	C603	NP_112442.2	heat shock protein 8	6.3	6.4
VCRTWLYNLK	C1217	XP_00148138 0.1	PREDICTED: doublecortin domain containing 5	4.6	2.6
VEDMAELTCLNEASVL HNLR	C95	NP_038635.1	myosin, heavy polypeptide 11, smooth muscle	12.7	22.4
VGELCAGK	C313	NP_033528.3	vinculin	40.3	62.2
VGINDFCPMGFGVK	C322	NP_031568.2	biglycan	65.2	45.7
VGTECGNQK	C574	NP_034357.2	filamin, alpha	27.8	31.8

VGVNDFCPTVPK	C326	NP_079987.2	asporin	36.6	51.6
VHSPSGALEECYVTEIDQDK	C2370	NP_034357.2	filamin, alpha	19.5	53.7
VLHDGGCSLPILR	-	NP_783572.2	latent transforming growth factor beta binding protein 4 isoform a	74.4	96.4
VPFLVLECPNLK	C14	NP_079785.1	DC2 protein	11.1	15.8
VPSGLYLGTCER	C1156	NP_032331.2	perlecan (heparan sulfate proteoglycan 2)	61.4	89.9
VPTPNVSVVDLTCR	C245	XP_001473992.1	PREDICTED: similar to Glyceraldehyde-3-phosphate dehydrogenase (GAPDH) isoform 1	11.9	25.9
VSHALAEGLGVIACIGEK	C127	NP_033441.1	triosephosphate isomerase 1	40.2	37.8
VTYCPTEPGNYIINIK	C2099	NP_034357.2	filamin, alpha	41.1	61.9
VVQCSDLGLDK	C62	NP_031859.1	decorin	45.8	53.9
VVQCSDLGLK	C77	NP_031568.2	biglycan	36.5	49.3
YGISLCQAILDETK	C324	NP_034860.2	annexin A1	52.6	21.1
YLIVVTDGHPLEGYKEPCGGLEDAVNEAK	C168	NP_034063.1	collagen, type VI, alpha 1	72.4	74.7
YSGCLTESNLIK	C553	NP_033399.1	transglutaminase 2, C polypeptide	33.3	32.3
YTGHHAYASGCTISPY	-	NP_034858.2	lysyl oxidase	88.4	89.1
YVICVR	C1715	NP_034357.2	filamin, alpha	34.7	24.3
